# Inappropriate antibiotic prescribing in Ethiopian hospitals: a systematic review

**DOI:** 10.1007/s11096-026-02157-5

**Published:** 2026-05-12

**Authors:** Abdella Birhan Yabeyu, Sarah Wise, Andrew Hayen, Jane Ellen Carland

**Affiliations:** 1 School of Public Health, Faculty of Health, University of Technology Sydney, Sydney, Australia; 2 School of Clinical Medicine, St Vincent’s Healthcare Clinical Campus, Faculty of Medicine and Health, University of New South Wales Sydney, Sydney, Australia; 3 Department of Clinical Pharmacology and Toxicology, St Vincent’s Hospital, Sydney, Australia

**Keywords:** Anti-bacterial agents, Antibiotic prescribing, Drug utilization review, Ethiopia, Hospitals, Inappropriate prescribing

## Abstract

**Introduction:**

Inappropriate antibiotic prescribing is a major driver of antimicrobial resistance globally, and its consequences are more severe in low- and middle-income countries such as Ethiopia. Although antibiotics are commonly prescribed for inpatients in Ethiopian hospitals, evidence on their appropriateness remains limited and inconsistent.

**Aim:**

This systematic review aimed to assess the prevalence, prescribing characteristics, and factors associated with inappropriate antibiotic prescribing in Ethiopian hospitals.

**Method:**

We searched MEDLINE, Embase, Scopus, Web of Science from inception to 4 May 2025, and also searched Google Scholar and manually reviewed the reference lists of eligible studies. Primary studies conducted in Ethiopian hospitals that reported on the appropriateness of inpatient antibiotic prescribing were included. Two authors independently screened studies and extracted data. Methodological quality was assessed using Joanna Briggs Institute critical appraisal tools. Because of the heterogeneity in study population, definitions and outcome measures, findings were synthesised descriptively. The review was registered in PROSPERO (CRD420251051881).

**Results:**

Fifty-three studies were included from seven Ethiopian regions, most from Amhara (n = 16). Overall, study quality was variable, with common limitations including unclear sampling and incomplete methodological reporting. Most studies focused on adult patients (n = 24) and used the Ethiopian Standard Treatment Guideline (n = 32). Cephalosporins were most frequently assessed (n = 45), ceftriaxone the most commonly evaluated antibiotic (n = 44). Prophylactic and empiric prescribing were commonly reported (n = 29 and n = 28) respectively. The prevalence of inappropriate prescribing reported per patient was a median of 56.8% (range, 10.2% to 91.4%) in 43 studies, and per prescription was a median of 40.7% (range, 19.2% to 66.9%) in nine studies. Indication, dose, dosing frequency, and duration were the prescribing characteristics most frequently reported. Patient-related factors, including comorbidity and polypharmacy were frequently associated with inappropriate prescribing.

**Conclusion:**

Inappropriate antibiotic prescribing was common in Ethiopian hospitals, although prevalence varied widely. Inconsistencies in guideline use and prescribing criteria likely contribute to this variability. Frequent empiric and prophylactic prescribing and reliance on ceftriaxone may reflect limited diagnostic capacity and raise concerns about antimicrobial resistance. Strengthening antimicrobial stewardship through standardised prescribing audits and improved diagnostic services is urgently needed to support appropriate antibiotic use in Ethiopia.

**Supplementary Information:**

The online version contains supplementary material available at 10.1007/s11096-026-02157-5.

## Impact statement

- This review demonstrates that inappropriate antibiotic prescribing is common across Ethiopian hospitals, highlighting the need to strengthen antimicrobial stewardship to improve prescribing quality and patient outcomes.
- The findings show a heavy reliance on empiric and prophylactic antibiotic use due to limited diagnostic capacity, emphasising the importance of expanding access to microbiology services to support more evidence-informed prescribing practice.
- The predominant use of broad-spectrum antibiotics particularly ceftriaxone indicates a critical need for targeted stewardship strategies to preserve antibiotic effectiveness and reduce resistance risks.
- Variability and inconsistencies in how Ethiopia’s standard treatment guidelines are applied highlight the need for standardised prescribing audits to promote more consistent, guideline-aligned practice across hospitals.

## Introduction

Antibiotics are among the most commonly prescribed medicines worldwide [1] yet inappropriate prescribing remains common across diverse resource settings [2]. Such misuse is associated with poor treatment outcomes, prolonged hospital stays and higher rates of rehospitalisation, all of which contribute to increased healthcare costs. Of growing concern, inappropriate antibiotic prescribing is also recognised as a major driver of the emergence and spread of antimicrobial resistance (AMR) [3–6]. The acceleration of AMR is now recognised as a leading global public health threat [7]. According to the World Health Organization (WHO), millions of deaths each year are attributed to AMR, with projections suggesting up to 10 million deaths annually by 2050 if urgent action is not taken [8].

Inappropriate prescribing of antibiotics includes use without a valid clinical indication, incorrect dose, frequency, or duration of therapy [9, 10]. In high-income countries, it has been linked to health inequalities, with higher antibiotic use observed among older adults, women, lower-income groups, and populations living in areas of greater deprivation [11]. This is amplified in low- and middle-income countries (LMICs) by structural challenges, including limited diagnostic capacity, inconsistent guideline implementation, and weak antimicrobial stewardship (AMS) programs [12, 13].

In Ethiopia, inappropriate antibiotic prescribing appears to be widespread [14–18], exacerbated by empiric therapy, where treatment is initiated before the causative pathogen and its drug susceptibility have been confirmed by microbiological testing [19, 20]*.* Inadequate training and limited awareness of guideline recommendations among healthcare professionals further contribute to frequent deviations from recommended practice [19, 21]. To address these challenges, the Ethiopian government introduced the AMR Prevention and Containment Strategic Plan (2021–2025) [22]. However, insufficient funding, workforce shortages, weak enforcement, and poor coordination across sectors have hampered implementation [23]. A systematic review in West Africa reported appropriate antibiotic prescribing ranged from 2.5 to 93%, highlighting inconsistent prescribing practice across settings [24]. Similarly, in Ethiopia, about 49% of antibiotic use was found to be inappropriate across mixed healthcare settings [25], but these studies did not examine inpatient, guideline-based prescribing, the key evidence gap this review seeks to fill.

One approach to promote better antimicrobial prescribing is the use of standardised national prescribing audits. Programs such as the National Antimicrobial Prescribing Survey in Australia [26] and the English Surveillance Programme for Antimicrobial Utilisation and Resistance in the United Kingdom [27], systematically assess prescribing practices and provide structured feedback on prescribing quality. These data help identify areas for improvement and encourage the uptake of AMS programs. Pharmacists play a key role in AMS activities, through audit and feedback interventions, where they support optimisation of antimicrobial therapy and promote adherence to clinical guidelines [28]. In Ethiopia, however, evidence on the magnitude and patterns of inappropriate prescribing is fragmented, consisting of a patchwork of individual studies, that vary widely in scope, methodology, and outcome measures. This systematic review sought to address this fragmented evidence base by undertaking the first comprehensive synthesis of studies examining inappropriate antibiotic prescribing in Ethiopian hospitals, with a specific focus on inpatient settings and prescribing appropriateness.

## Aim

This systematic review aimed to assess the prevalence, prescribing characteristics, and factors associated with inappropriate antibiotic prescribing in Ethiopian hospitals.

## Method

### Protocol and registration

This systematic review was reported according to the Preferred Reporting Items for Systematic Reviews and Meta-Analyses (PRISMA) guideline 2020 [29]. The protocol is registered in PROSPERO (*CRD420251051881*)*.*

### Search strategy

Four electronic databases, MEDLINE, Embase, Scopus, and Web of Science, were searched from inception until 4 May 2025. Google Scholar was also searched and reference lists from eligible articles were screened. We restricted the search to studies conducted in Ethiopian hospitals that reported on the appropriateness of antibiotic prescribing among inpatients. This reflects the primary target of Ethiopian AMS activities and prescribing audits. We used a combination of exploded MeSH terms and free-text keywords, with Boolean operators used to combine terms (Supplementary Table 1). Search terms covered four areas: antibiotics, prescribing practices, hospital settings, and Ethiopia.

### Study selection and review process

Two authors (ABY, JEC) independently screened titles and abstracts and reviewed full texts deemed potentially eligible. Any disagreements were resolved through discussion, with input from a third author when required. Studies were eligible if they reported the prevalence of inappropriate antibiotic prescribing among inpatients in Ethiopian hospitals based on any stated approach to assessing appropriateness (e.g., guideline-based or other defined criteria), without restriction on the type or number of parameters used. We included studies irrespective of the population studied (paediatrics or adults), the drug class or study design. We excluded studies that were not primary research, if the full text was not available, or if they were conducted in outpatient or community settings.

### Data extraction and synthesis

Two authors (ABY, JEC) independently extracted data from eligible studies using a custom Excel data collection sheet. Extracted variables included study characteristics (e.g., sample size, study duration), hospital characteristics (e.g., patient population, geographic region), antibiotics assessed (drug and drug class), hospital department(s) involved, reasons for antibiotic use, guideline(s) used to evaluate prescribing, prevalence of inappropriate prescribing, prescribing characteristics (e.g., indication, dose), factors associated with inappropriate prescribing, and reported study limitations.

The prevalence of inappropriate antibiotic prescribing was reported based on the denominator used to calculate it (per patient, per prescription, or not reported). Prevalence estimates were summarised descriptively across studies, with each study contributing an estimate, as the objective was to describe the distribution of reported values rather than weighted sample size.

Given substantial heterogeneity in definitions of inappropriate prescribing, studies were classified according to the parameters used to assess appropriateness. Specifically, studies were grouped into: (1) those assessing inappropriate prescribing based on one or more prescribing parameters (indication, dose, dosing frequency, and/or duration of therapy); (2) those assessing these prescribing parameters in combination with additional variables (e.g., availability of culture testing, adverse drug reactions, drug interactions, inappropriate drug choice, non-adherence, timing or route of administration, or need for additional therapy); and (3) those that did not clearly report the criteria used. These classifications were used to characterise heterogeneity in outcome definitions and reporting and to inform interpretation of the findings.

### Risk of bias assessment

Two authors (ABY, JEC) independently assessed the methodological quality of included studies using the Joanna Briggs Institute critical appraisal checklists, as appropriate for each study design. The checklist is comprised of eight items for cross-sectional studies, eleven items for cohort studies, and nine items for the quasi-experimental studies. Responses to each item were recorded and the findings were synthesised narratively.

## Results

**Fig. 1:**
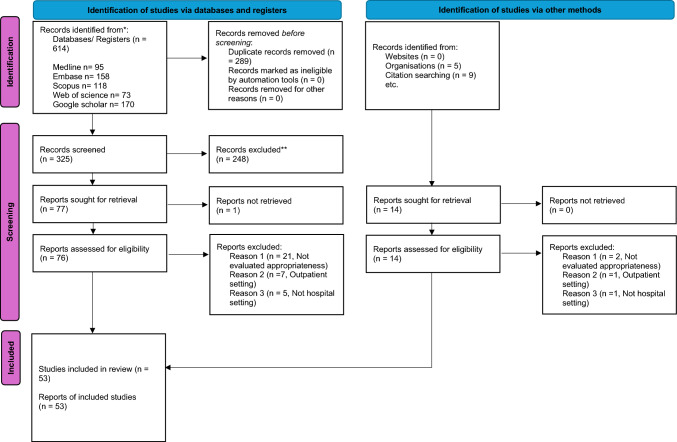
PRISMA flow diagram of study selection

We identified 614 records through database searches and an additional nine through citation searching, of which we included 53 studies in the final review (Fig. 1). The studies were published between 2012 and 2025 and were undertaken in seven Ethiopian regions, most in the Amhara (n = 16) [15, 30–44] and Oromia (n = 13) [6, 16, 45–55] regions. Most studies were conducted at a single site (n = 48) [6, 15, 16, 30–46, 48–75], commonly a university or tertiary referral hospital (n = 37) [6, 15, 16, 30–37, 39, 41–45, 48–51, 54–66, 68–70], and most were conducted in medical wards (n = 32) [16, 30, 32–37, 42, 43, 47, 48, 51–53, 55–57, 59–65, 67, 70, 72, 73, 75–77]. The majority (n = 41) [15, 30, 31, 33–35, 37–43, 45, 46, 49–54, 56, 58, 59, 61, 63–67, 69–79] employed a cross-sectional design, eleven were cohort studies [6, 16, 32, 36, 44, 47, 48, 55, 57, 62, 68], and one was quasi-experimental [60]. The data collection period ranged from one [71, 78] to 50 months [44]. Most studies captured data for adult patients (≥ 18 years) (n = 24), [16, 30, 32–37, 43, 47, 48, 52–55, 57, 61, 63–65, 70, 71, 73, 77] with sample sizes ranging from 84 [37] to 1,079 [67] patients (Table 1).

**Table 1:** Study characteristics

Author/s, year and region	Study design	Population (Sample size)	Study period	Drug class (a) (Drug/s) (b)	Guideline/Criteria (c)
Abebe et al. [56], Tigray	Cross-sectional	Adult & paediatric (n = 296)	Jul 2011 to Sep 2011	CEP (CRO)	ETSG
Adere et al. [57], SNNP	Prospective cohort	Adult (n = 128)	Jun 2021 to Aug 2021	CEP, FQ, MAC (AZM, CRO, CIP)	ESTG, WHO guideline, IDSA
Adugna et al. [30], Amhara	Cross-sectional	Adul (n = 265)	May 2022 to Jul 2022	GLY (VAN)	ESTG
Afework et al. [58], Addis Ababa	Cross-sectional	Adult & paediatric (n = 220)	Jun 2018 to Jun 2021	Not specified	No guideline
Alekaw et al. [31], Amhara	Cross-sectional	Paediatric (n = 279)	Sep 2020 to Aug 2021	AG, CEP, FQ, GLY, MAC, PEN (AMX, AMP, CRO, CLX, CPEN, AMP + CAZ, AMP + CRO + GEN, AMP + CRO + MTZ, AMP + CRO, AMP + GEN + CRO, AMP + GEN + MTZ, AMP + GEN, CRO + CEX, CRO + MTZ, CRO + VAN, CIP + VAN + MTZ, MTZ + VAN)	ESTG, WHO guideline
Amare et al. [76], Harari	Cross-sectional	Adult & paediatric (n = 271)	Jan 2016 to Dec 2016	CEP (CRO)	ESTG
Anteneh et al. [32], Amhara	Prospective cohort	Adult (n = 303)	Mar 2020 to Jun 2020	AG, CEP, FQ, GLY, MAC, PEN (AMP, AZM, CEX, CAZ, CRO, CIP, SXT, GEN, MTZ, VAN)	ESTG, IDSA
Asefa et al. [45], Oromia	Cross-sectional	Paediatric (n = 341)	Feb 2013 to May 2013	AG, CEP, MAC, PEN, SUL, TTC (AMX, AMC, AMP, AZM, BEN-PEN, CRO, CEX, CHL, CIP, CLX, SXT, CPEN, DOX, ERY, GEN, NOR, PROC-PEN)	ESTG
Ayele et al. [33], Amhara	Cross-sectional	Adult (n = 390)	Jan 2017 and Mar 2017	CEP (CRO)	ESTG, ASHSP
Ayinalem et al. [34], Amhara	Cross-sectional	Adult (n = 316)	Dec 2011 to Jan 2013	CEP (CRO)	ESTG
Bantie [35], Amhara	Cross-sectional	Adult (n = 264)	Mar 2013 to May 2013	CEP (CRO)	ESTG
Firomsa et al. [46], Oromia	Cross-sectional	Paediatric (n = 248)	Jan 2018 to Feb 2018	AG, AMPH, CEP, PEN, SUL, TTC (AMX, AMP, CRO, CEX, CHL, CLX, SXT, CPEN, GEN)	EPDF, WHO
Firomsa et al. [48], Oromia	Prospective cohort	Adult (n = 172)	May 2019 to Aug 2019	AG, CEP, GLY, MAC, SUL (CRO, SXT, GEN, VAN)	No guideline
Firomsa et al. [48] , Oromia	Prospective cohort	Adult (n = 313)	Mar 2020 to Jun 2020	CEP, NI, PEN, TTC (AMC, CRO, DOX, MTZ)	ESTG
Belayneh et al. [36], Amhara	Prospective cohort	Adult (n = 147)	Mar 2014 to May 2014	Not specified	CDRPC
Birarra et al. [37], Amhara	Cross-sectional	Adult (n = 84)	Mar 2018 to May 2018	Not specified	DPRFDGAD
Debela et al. [16], Oromia	Prospective cohort	Adult (n = 300)	Oct 2020 to Apr 2021	AG, CARB, CEP, FQ, GLY, MAC, PEN (AMC, AMP, AZM, CAZ, CRO, CEX, CIP, DOX, ERY, GEN, MEM, MTZ, NOR, VAN)	ESTG, IDSA, SSIPG, CAUTI
Fekadu et al. [49], Oromia	Cross-sectional	Paediatric (n = 124)	Mar 2013 to Apr 2013	AG, AMPH, CEP, NI, PEN, SUL (AMX, AMC, AMP, CRO, CEX, CHL, CLX, SXT, CPEN, GEN, MTZ, PROC-PEN, AMP + GEN, CRO + GEN, CEX + GEN, CHL + CPEN, CLX + CHL, CLX + GEN)	EPDF, WHO guideline
Garedow and Tesfaye [50], Oromia	Cross-sectional	Paediatric (n = 402)	Apr 2021 to Nov 2021	AG, AMPH, CEP, FQ, GLY, MAC, NI, PEN (AMX, AMP, CAZ, CRO, CHL, CLX, GEN, MTZ, AZM, AMP + GEN + MTZ, AMP + GEN, CRO + AMP, CRO + GEN + CLX, CRO + GEN, CRO + MTZ, PEN + CHL)	ESTG, WHO Guideline
Gashaw et al. [51], Oromia	Cross-sectional	Adult & paediatric (n = 384)	Feb 2019 and Mar 2019	AG, AMPH, CEP, FQ, GLY, MAC, NI, PEN, SUL, TTC (AMX, AMC, AMP, AZM, CEX, CAZ, CRO, CHL, CIP, CLR, CLX, SXT, DOX, GEN, MTZ, NOR, VAN)	ESTG
Gebremichael et al. [59], Tigray	Cross-sectional	Adult & paediatric (n = 327)	Feb 2019 to Apr 2019	CEP (CAZ)	CCDUECPI
Gebretekle et al. [60], Addis Ababa	Quasi-experimental	Adult & paediatric (n = 707)	Nov 2017 to Jan 2019	Not specified	Institutional-based guidelines
Geresu et al. [61], SNNP	Cross-sectional	Adult (n = 403)	Mar 2012 to Mar 2015	CEP (CRO)	ESTG
Gidey et al. [63], Tigray	Cross-sectional	Adult (n = 422)	Aug 2012 to Feb 2013	Not specified	No guideline
Gidey et al. [62], Tigray	Prospective cohort	Paediatric (n = 232)	Sep 2019 to Nov 2019	AG, CARB, CEP, FQ, GLY, MAC, PEN (AMX, AMP, AZM, CAZ, CRO, CIP, SXT, GEN, MEM, MTZ, VAN)	CDRPC
Gube et al. [52], Oromia	Cross-sectional	Adult (n = 200)	Mar 2016 to May 2016	AG, CEP, FQ, MAC, PEN, TTC (AMC, AMP, CRO, CEX, CHL, CIP, CLR, CLX, SXT, DOX, GEN, MTZ, NOR)	No guideline
Habteweld et al. [15], Amhara	Cross-sectional	Adult & paediatric (n = 204)	Oct 2022 to Jan 2023	CEP, NI, PEN (AMP, CRO, MTZ)	ESTG, CPGAPS
Jambo et al. [78], Harari	Cross-sectional	Adult & paediatric (n = 693)	May 1 and 31, 2021	AG, AMPH, CEP, FQ, GLY, MAC, NI, PEN (CRO, CPEN, AMP + GEN, CRO + AMP, CRO + AZM, CRO + CLX, CRO + MTZ, CRO + VAN, VAN + FEP, VAN + CAZ)	ESTG, IDSA, ATS
Jifar et al. [53], Oromia	Cross-sectional	Adult (n = 185)	Jul 2021 and Dec 2021	CEP (CRO)	ESTG
Kebede et al. [75], Dire Dawa	Cross-sectional	Adult & paediatric (n = 174)	Dec 2017 to Jan 2018	CEP (CRO)	ESTG
Kefale et al. [38], Amhara	Cross-sectional	Adult & paediatric (n = 281)	Jun 2020 to Jul 2020	AG, CEP, FQ, NI, PEN (CRO, CIP, CLX, CRO + GEN + MTZ, CRO + MTZ)	ESTG, WHO guideline, ASHSP
Ketema et al. [64], Addis Ababa	Cross-sectional	Adult (n = 264)	Mar 2016 to Apr 2016	AG, CARB, CEP, FQ, GLY, MAC, NI, PEN, TTC (CIP, CLX, SXT, MEM, NIT, VAN, AMP + GEN, FEP + VAN, CRO + AZM, CRO + CLX, CRO + SXT + AZM, CRO + GEN, CRO + MTZ + CIP, CRO + VAN + AMP, CRO + VAN + SXT, CRO + VAN + MTZ, CRO + VAN, CIP + CLIN, CIP + CLX, CIP + MTZ, CLX + GEN, MEM + CLIN, MEM + MTZ, MTZ + NOR, VAN + MEM, VAN + MTZ)	IDSA
Mama et al. [54], Oromia	Cross-sectional	Adult (n = 471)	Sep 2018 to Apr 2019	CEP, FQ, GLY, MAC, NI, PEN, TTC (AMC, AMP, AZM, CRO, CIP, CLX, DOX, ERY, MTZ, VAN)	ESTG, WHO guideline, ASHSP
Mehari [65], Addis Ababa	Cross-sectional	Adult (n = 300)	Jul 2015 and Sep 2015	CEP (CRO)	ESTG, ASHSP, institution-based guideline
Mengesha and Mohammed [39], Amhara	Cross-sectional	Paediatric (n = 157)	Mar 2022 and Apr 2022	AG, CEP, FQ, GLY, MAC, PEN (AMX, AMC, AZM, CRO, AMP + GEN, CAZ + VAN, CRO + MTZ, CRO + VAN)	ESTG
Moges et al. [40], Amhara	Cross-sectional	Adult & paediatric (n = 227)	Apr 2017 to Mar 2019	CEP, FQ, NI, PEN (CRO, CIP, CLX, MTZ + CRO, MTZ + CEX)	WHO guideline, ASHSP
Mohamoud et al. [66], Tigray	Cross-sectional	Adult & paediatric (n = 196)	Mar 2015 to Apr 2015	CEP, NI, PEN (AMP, CRO, AMX + MTZ, CRO + MTZ)	ESTG, ASHSP
Muhammed and Nasir [67], Addis Ababa	Cross-sectional	Adult & paediatric (n = 1,079)	May 2017 to Apr 2018	CEP (CRO)	ESTG
Mulat [41], Amhara	Cross-sectional	Adult & paediatric (n = 412)	Jan 2023 to Aug 2023	CEP, NI (CFZ, CRO, CIP, MTZ + CFZ, MTZ + CRO, MTZ + CIP)	WHO guideline
Niriayo et al. [68], Tigray	Prospective cohort	Adult & paediatric (n = 272)	Dec 2018 to Apr 2019	CEP, FQ, GLY, MAC, PEN (AMX, CAZ, CRO, CEX, CIP, CLX, GEN, MTZ, VAN)	CDRPC
Sewagegn et al. [42], Amhara	Cross-sectional	Adult & paediatric (n = 127)	Apr 2015 and Jun 2015	CEP (CRO)	WHO guideline
Shegute et al. [79], Tigray	Cross-sectional	Adult & paediatric (n = 800)	Dec 2015 to Aug 2016	CEP (CRO)	ESTG
Shimels and Fenta [69], Addis Ababa	Cross-sectional	Adult & paediatric (n = 571)	May 2013 to Apr 2014	CEP (CRO)	ESTG, IDSA, ASHSP
Shimels et al. [77], Addis Ababa	Cross-sectional	Adult (n = 477)	Jan 2014 to Feb 2014	CEP (CRO)	ESTG
Sileshi et al. [70], Addis Ababa	Cross-sectional	Adult (n = 314)	Feb 2014 and Jun 2014	CEP (CRO)	ESTG, WHO guideline, SGAT
Taressa et al. [71], Harari	Cross-sectional	Adult (n = 215)	Jan 2018 to Dec 2019	CEP (CRO)	WHO guideline
Tassew et al. [72], Addis Ababa	Cross-sectional	Adult & paediatric (n = 410)	Mar 2017 to Aug 2017	Not specified	IDSA
Tefera et al. [6], Oromia	Prospective cohort	Adult & paediatric (n = 300)	Apr 2017 to Jul 2017	AG, CEP, FQ, NI, PEN (AMP, CAZ, CRO, CHL, CIP, CLX, CPEN, MTZ, AMP + CHL, CRO + MTZ)	ESTG, WHO Guideline, IDSA, ASHSP
Tessema et al. [43], Amhara	Cross-sectional	Adult (n = 130)	Jan 17 to Jan 25 2011	AG, CEP, FQ, MAC, NI, PEN, TTC, SUL (AMC, AMX, AMP, CRO, CIP, CLR, CLX, SXT, CPEN, DOX, ERY, GEN, AMX + CLR, AMP + GEN, CRO + CLX, CRO + GEN, CPEN + GEN)	ESTG
Werede et al. [73], Addis Ababa	Cross-sectional	Adult (n = 399)	Mar 2019 to Feb 2020	CEP (CRO)	No guideline
Wondm et al. [44], Amhara	Retrospective cohort	Adult & paediatric (n = 416)	Jan 2017 to Feb 2021	CARB, CEP, GLY, NI (FEP, CAZ, FEP + VAN, CAZ + VAN, MEM + VAN, MTZ + CRO)	IDSA
Yadesa et al. [55], Oromia	Prospective cohort	Adult (n = 152)	Feb 2014 to Mar 2014	Not specified	IDSA
Yehualaw et al. [74], Tigray	Cross-sectional	Paediatric (n = 692)	Dec 2018 to Apr 2019	AG, AMPH, CEP, FQ, GLY, MAC, NI, PEN (AMX, AMP, CRO, CHL, CIP, CLR, CLX, SXT, MTZ, CRO + AMX, AMP + GEN, CRO + MTZ, MTZ + CLX, CRO + CLX + MTZ, CRO + AMC, CRO + GEN, CRO + CIP, CRO + CEX, CLX + GEN, CRO + CHL, CRO + CLX, AMP + CIP)	ESTG, WHO Guideline

(a)CEP: Cephalosporin, MAC: macrolide, FQ: fluoroquinolone, AM: aminoglycoside, PEN: penicillin, AMP: ampicillin, SUL: sulfonamide, TTC: tetracycline, CARB: carbapenem

(b)CRO: Ceftriaxone, AZM: Azithromycin, CIP: ciprofloxacin, AMX: amoxicillin, CLX: cloxacillin, CPEN: crystalline penicillin, CAZ: ceftazidime, GEN: gentamicin, MTZ: metronidazole, CEX: cephalexin, SXT: cotrimoxazole, AMC: amoxicillin/clavulanic acid, BEN-PEN: benzathine penicillin, CHL: chloramphenicol, DOX: doxycycline, ERY: erythromycin, NOR: norfloxacin, PROC-PEN: procaine penicillin fortified, AMPH: amphenicol, NI: nitroimidazole, MEM: meropenem, CLR: clarithromycin, FEP: cefepime, NIT: nitrofurantoin, CLIN: clindamycin, CFZ: Cefazolin

(c)ESTG: Ethiopian Standard Treatment Guideline, IDSA: Infectious Diseases Society of America, ASHSP: American Society of Health System Pharmacist’s Guideline, EPDF: Ethiopian Paediatric Drug Formulary, CDRPC: Cipolle’s drug-related problems classification, DPRFDGAC: Drug Prescribing in Renal Failure: Dosing Guidelines for Adults and Children, SSIPG: Surgical Site Infection Prevention Guideline, CAUTI: Catheter-Associated Urinary Tract Infections, CCDUECPI: Concurrent ceftazidime drug use evaluation with clinical pharmacy intervention, CPGAPS: Clinical practice guidelines for antimicrobial prophylaxis in surgery, ATS: American Thoracic Society, SGAT: Sanford Guide to Antimicrobial Therapy

### Risk of bias and study quality

Across studies, most of them reported the eligibility criteria and used appropriate outcome measures. However, several methodological limitations were common. In particular, many studies had unclear or non-random sampling, insufficient identification or handling of confounding (limited description of confounders or strategies to address them). Incomplete reporting of method details also resulted in multiple “Unclear” responses (Supplementary Table 2).

### Antibiotics prescribing guidelines

Of the 53 studies, 48 reported using at least one guideline to assess prescribing appropriateness. Among these, 29 relied on a single guideline, while 19 used multiple guidelines [6, 15, 16, 31–33, 38, 40, 46, 49, 50, 54, 57, 65, 66, 69, 70, 74, 78]. The Ethiopian Standard Treatment Guideline was the most frequently cited (n = 32) [15, 16, 30–35, 38, 39, 43, 45, 47, 50, 51, 53, 54, 56, 57, 61, 65–67, 69, 70, 74–79]. Five studies did not report using any guideline [48, 52, 58, 63, 73].

### Antibiotics assessed

Nineteen studies evaluated prescribing of a single antibiotic [30, 33–35, 42, 53, 56, 59, 61, 65, 67, 69–71, 73, 75–77, 79], of which 17 focused on ceftriaxone, with one study each assessing ceftazidime and vancomycin. While 27 studies assessed multiple antibiotics [6, 15, 16, 31, 32, 36, 38–41, 43–52, 54, 57, 62, 64, 66, 68, 74, 78]. Seven studies did not specify the type and number of antibiotics assessed [36, 37, 55, 58, 60, 63, 72]. In total, 30 different antibiotics were reported. Cephalosporins were the most frequently evaluated drug class (n = 45), followed by penicillins (n = 23) and aminoglycosides (n = 18). At the individual drug level, ceftriaxone prescribing was reported in 44 studies.

### Indications for antibiotic use

The indication for antibiotic prescribing was categorised as prophylactic, empiric, or definitive (culture- and sensitivity-guided). Empiric prescribing was reported in 28 studies [6, 30–33, 39, 45, 49–54, 56, 59, 62, 64, 65, 67, 69–73, 75, 77–79], with the median proportion of empiric prescriptions across studies being 82.1% (range, 55.4–100%) and most commonly reported in medical (n = 13) wards. Prophylaxis (n = 29) [6, 15, 30, 31, 33, 38–41, 45, 49–54, 56, 58, 62, 64–67, 69, 71, 73, 75, 77, 79], accounted for a median of 27.0% of antibiotic prescriptions (range, 0.4–100.0%) and most commonly reported in surgical wards (n = 14). Culture- and sensitivity-guided prescribing was reported in 13 studies [31–33, 45, 49, 50, 52, 59, 65, 70–72, 79], where it represented a median of 7.4% (range, 1.6–23.2%). Culture-guided prescribing was most reported in medical (n = 6) and paediatric (n = 6) wards. Nineteen studies did not specify indication [16, 34–37, 42–44, 46–48, 55, 57, 60, 61, 63, 68, 74, 76].

### Prevalence of inappropriate antibiotic prescribing

The measure of inappropriate prescribing varied significantly across studies. Seventeen studies assessed inappropriate prescribing based on one or more prescribing parameters, including indication, dose, dosing frequency, and/or duration of therapy, although not all studies included all parameters. Twenty-six studies assessed these parameters in combination with additional variables such as availability of culture testing, adverse drug reactions, drug interactions, inappropriate drug choice, non-adherence, timing or route of administration, or need for additional therapy. The additional variables were inconsistently applied across studies, leading to substantial variation in the types and numbers of parameters included. Ten studies did not clearly report the criteria used.

**Fig. 2:**
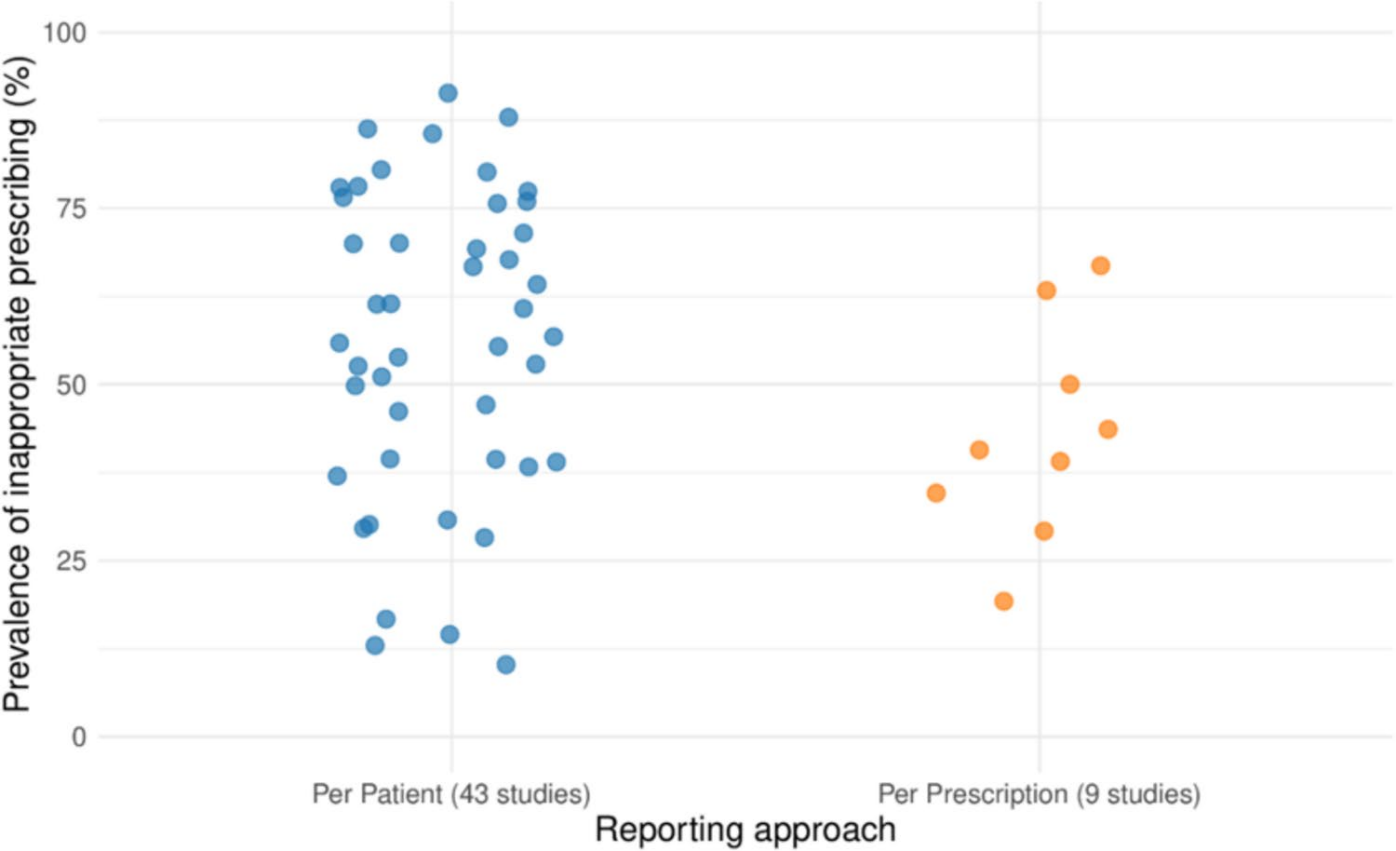
Prevalence estimates of inappropriate prescribing

The prevalence and characteristics of inappropriate antibiotic prescribing varied widely. Most studies quantified inappropriate prescribing at either the patient or prescription level. Forty-three studies [6, 15, 16, 31–35, 38–42, 44, 47, 48, 51–58, 61–79] reported the prevalence per patient, with a median of 56.8% (range, 10.2% to 91.4%). Nine studies [30, 36, 37, 45, 46, 49, 50, 59, 60] reported prevalence per prescription, with a median of 40.7% (range, 19.2–66.9%) (Fig. 2). Per patient estimates tended to be higher than those calculated per prescription. One study did not report a prevalence estimate [43].

Studies conducted across seven Ethiopian regions, revealed substantial geographic variability. In Amhara region, prevalence ranged from 10.2 to 91.4% (n = 16); in Oromia, from 14.5 to 76.0% (n = 13); in Addis Ababa, from 39.4 to 87.9% (n = 10); in Tigray, from 28.3 to 78.1% (n = 8); in Harari, from 16.7 to 70.1% (n = 3); and in Southern Nations, Nationalities, and People’s Region, 60.8% and 76.6% (n = 2). A single study in Dire Dawa reported a prevalence of 80.5%.

### Characteristics of inappropriate antibiotic prescribing

Forty-three studies evaluated specific characteristics of inappropriate prescribing. Most assessed one of more of the following parameters: clinical indication, dose, dosing frequency, and duration of therapy.

*Inappropriate indication,* defined as prescribing an antibiotic without a valid, evidence-based clinical need according to the guidelines, was reported in 33 studies [6, 15, 16, 30, 31, 33–35, 38, 40–43, 48–50, 53, 55, 57–59, 62, 65–68, 70, 71, 74–77, 79].

*Inappropriate dose* was defined as prescribing antibiotics above or below the guideline recommendation for a given indication or patient group. Thirty-four studies [6, 16, 30–35, 37, 38, 40, 42, 45–47, 50, 52–55, 57, 59, 61, 62, 65–68, 70, 71, 74, 76, 77, 79] assessed dosing errors, of which 11 [6, 35, 37, 46, 47, 55, 57, 62, 66, 68, 74] specified whether the dose was too high or too low.

*Inappropriate dosing frequency* was defined as deviations from guideline recommended dosing intervals. Twenty-two studies [16, 30, 31, 33, 34, 42, 45, 46, 49, 50, 52, 53, 59, 61, 65, 67, 70, 74–77, 79] reported errors in dosing frequency. Of these, three specified whether dosing was more frequent or less frequent than the recommendation [46, 49, 74].

*Inappropriate duration* was defined as prescribing a course of an antibiotic that was longer or shorter than the guideline recommendation. Thirty studies [15, 16, 30, 31, 33–35, 40–42, 45, 46, 49, 50, 52, 53, 58, 59, 61, 65, 67, 70–72, 74–79] assessed duration of antibiotic treatment. Among these, seven studies reported how the course of therapy deviated from guidelines [16, 35, 46, 49, 58, 72, 78].

Several studies reported additional indicators, including inappropriate drug selection or ineffective therapy (n = 14) [6, 16, 38, 40, 41, 47, 48, 54, 55, 57, 62, 68, 72, 78], need for additional antibiotics (n = 8) [6, 47, 48, 55, 57, 62, 66, 68], drug-drug interactions (n = 7) [31, 33, 35, 42, 59, 65, 70], adverse drug reactions (n = 7) [6, 47, 48, 55, 57, 62, 68], non-adherence to the prescribed antibiotics (n = 7) [6, 47, 48, 55, 57, 62, 68] and absence of culture and sensitivity testing (n = 4) [32, 33, 65, 70] (Table 2).

**Table 2:** Prevalence and characteristics of inappropriateness of antibiotic prescribing

Study	Reason for use	Prevalence (*%*)	Characteristics of inappropriate prescribing (%)
*Prevalence reported per patient*			
Abebe et al. [56]	Empiric 60.2%, Prophylaxis 39.8%	64.2	Not reported
Adere et al. [57]	Not specified	76.6	Adverse drug reactions = 26.5%, Dose too high = 9.2%, Dose too low = 15.3%, Ineffective drug therapy = 7.1%, Need for additional drug therapy = 36.7%, Non-adherence = 30.6%, Unnecessary use of antimicrobials (Indication) = 42.6%
Afework et al. [58]	Prophylaxis 100%	52.9	Indication = 23.8%, Duration (extended) = 76.2%
Alekaw et al. [31]	Empiric 88.9%, Definitive 2.3%, Prophylaxis 8.3%	30.8	Contraindication = 20.8%, Dose = 14.0%, Drug interactions = 12.9%, Duration = 16.1%, Frequency = 4.7%, Unnecessary use of antimicrobials (Indication) = 20.8%
Amare et al. [76]	Not specified	70.1	Dose = 1.6%, Duration = 28.4%, Frequency = 1.6%, Indication = 60.0%, Combination = 8.4%
Anteneh et al. [32]	Empiric 91.08%, Definitive 8.9%	91.4	No empirical antibiotics = 43.2%, Not according to the national guideline = 57.1%, No culture performed = 84.8%, No pathogen-directed therapy = 91.1%, No adapt antibiotics dosing = 44.9%, No intravenous to oral therapy = 72.9%
Ayele et al. [33]	Empiric 79.5%, Definitive 4.1%, Prophylaxis 16.4%	80.2	Dose = 22.3%, Drug-drug interaction = 15.9%, Duration = 58.8%, Frequency = 97.4%, Indication = 15.9%, No culture and sensitivity test = 85.6%
Ayinalem et al. [34]	Not specified	46.2	Dose = 22.6%, Duration = 47.3%, Frequency = 24.0%, Indication = 6.2%
Bantie [35]	Not specified	39.0	Dose too high = 8.2%, Dose too low = 6.0%, Drug interaction = 6.1%, Duration (extended) = 10.7%, Duration (short) = 7.4%, Indication = 8.2%
Firomsa et al. [48]	Not specified	67.7	Adverse drug reaction = 3.8%, Dose too high = 26.4%, Dose too low = 17.9%, Ineffective drug therapy = 14.2%, Need additional drug therapy = 23.6%, Non-adherence = 26.9%, Unnecessary use of antimicrobials (indication) = 43.4%
Debela et al. [16]	Not specified	76.0	Choice (Ineffective) = 44.1%, Dose = 38.1%, Duration (extended) = 8.8%, Frequency = 14.0%, Indication = 24.2%, Route = 3.5%
Gashaw et al. [51]	Empiric 70.3%, Prophylaxis 29.7%	52.6	Not reported
Geresu et al. [61]	Not specified	60.8	Duration = 82.0%, Dose = 12.2%, Frequency = 5.7%
Gidey et al. [63]	Not specified	55.9	Not reported
Gube et al. [52]	Empiric 81.5%, Definitive 16.0%, Prophylaxis 2.5%	14.5	Dose = 48.3%, Duration = 34.5%, Frequency = 17.2%
Habteweld et al. [15]	Prophylaxis 100%	78.0	Duration = 73.2%, Indication = 62.2%, Timing of administration = 76.8%
Jambo et al. [78]	Empiric 100%	16.7	Choice (Ineffective) = 46.6%, Duration (extended) = 53.4%
Jifar et al. [53]	Empiric 95.0%, Prophylaxis 5.0%	47.1	Dose = 10.3%, Duration = 49.4%, Frequency = 16.1%, Indication = 24.2%
Kebede et al. [75]	Prophylaxis 21.84%, Empiric 78.16%	80.5	Duration = 50.0%, Frequency = 20.71%, Indication = 29.29%
Kefale et al. [38]	Prophylaxis 100%	49.8	Choice (Ineffective) = 13.7%, Dose = 50.0%, Indication = 11.3%, Time of administration = 25.0%
Ketema et al. [64]	Prophylaxis 44.6%, Empiric 55.4%	61.5	Not reported
Mama et al. [54]	Prophylaxis 17.2%, Empiric 82.8%	30.1	Choice (Ineffective) = 39.4%, Dose = 60.6%
Mehari [65]	Empiric 62.7%, Definitive 3.3%, Prophylaxis 34.0%	85.6	Dose = 1.2%, Duration = 41.3%, Drug-drug interaction = 43.2%, Frequency = 18.3%, Indication = 56.03%, No culture = 17.5%
Mengesha and Mohammed [39]	Empiric 95.5%, Prophylaxis 16.9%	10.2	Not reported
Moges et al. [40]	Prophylaxis 100%	86.3	Choice (Ineffective) = 69.9%, Dose = 16.8%, Duration = 19.4%, Indication = 25.0%
Mohamoud et al. [66]	Prophylaxis 62.2%	78.1	Dose too high = 3.9%, Dose too low = 3.9%, Need additional drug = 20.3%, Unnecessary combination = 55.6%, Unnecessary use of antimicrobials (Indication) = 4.6%
Muhammed and Nasir [67]	Empiric 59.7%, Prophylaxis 39.8%	39.4	Dose/frequency = 4.2%, Duration = 8.4%, Indication = 36.7%, Unnecessary use of antimicrobials (Indication) = 50.6%
Mulat [41]	Prophylaxis 100%	77.4	Choice (Ineffective) = 88.8%, Duration = 42.4%, Indication = 9.8%, Timing of administration = 96.4%
Sewagegn et al. [42]	Not specified	70.0	Contraindication = 2.2%, Dose = 1.1%, Duration = 67.4%, Drug-drug interaction = 10.1%, Frequency = 12.4%, Indication = 6.7%
Shegute et al. [79]	KAH = Empiric 65.5%, Definitive 11.0%, Prophylaxis 23.5%: MGH = Empiric 93.8%, Definitive 4.2%, Prophylaxis 2.0%	KGH = 38.3%: MGH = 37.0%	KGH: Dose = 7.2%, Duration = 20.9%, Frequency = 0.7%, Indication = 71.2%: MGH: Dose = 16.2%, Duration = 29.1%, Frequency = 2.7%, Indication = 52.0%
Shimels and Fenta [69]	Empiric 60.6%, Prophylaxis 39.4%	39.4	Not reported
Shimels et al. [77]	ZGH = Empiric 91.5%, Prophylaxis 8.5%: HGH = Empiric 93.06%, Prophylaxis 6.9%	ZGH = 51.1%: HGH = 55.4%	Zewditu: Dose, route, frequency or duration of administration = (55.2%), Indication = (44.8%): Hayat: Dose, route, frequency or duration of administration = (39.3%), Indication = (60.7%)
Sileshi et al. [70]	Empiric 87.3%, Definitive 1.6%	87.9	Dose = 21.0%, Duration = 50.0%, Drug-drug interactions = 8.7%, Frequency = 80.3%, Indication = 18.5%, No culture = 53.2%
Taressa et al. [71]	Empiric 80.5%, Definitive 19.1%, Prophylaxis 0.4%	13.0	Dose = 7.4%, Duration = 65.6%, Indication = 13.0%
Tassew et al. [72]	Empiric 94.1%, Definitive 5.9%	66.7	Choice (Ineffective) = 53.6%, Duration (extended) = 19.0%
Werede et al. [73]	Empiric 72.93%, Prophylaxis 27.06%	56.8	Not reported
Wondm et al. [44]	Not specified	29.6	Not reported
Yehualaw et al. [74]	Not Specified	28.3	Dose too high = 13.3%, Dose too low = 51.2%, Frequency (less) = 8.2%, Duration = 35.7%, Unnecessary use of antimicrobials (Indication) = 11.2%
*Prevalence reported per prescription*			
Adugna et al. [30]	Empiric 86.8%, Prophylaxis 13.2%	63.3	Dose = 35.8%, Duration = 24.5%, Frequency = 13.4%, Indication = 18.2%, Combination = 8.0%
Asefa et al. [45]	Empiric 83.58, Definitive 13.49%, Prophylaxis 2.93%	34.6	Dose = 48.1%, Duration = 30.2%, Frequency = 21.7%
Firomsa et al. [46]	Not specified	40.7	Dose too high = 22.1%, Dose too low = 4.1%, Duration (extended) = 12.3%, Duration (short) = 4.9%, Frequency (less) = 54.5%, Frequency (more) = 1.6%
Belayneh et al. [36]	Not specified	39.1	Not reported
Birarra et al. [37]	Not specified	50.0	Dose too high = (100%
Fekadu et al. [49]	Empiric 85.0%, Definitive 7.67%, Prophylaxis 7.33%	43.6	Duration (extended) = 0.54%, Duration (short) = 13.51%, Frequency (more) = 3.78%, Frequency (less) = 20.54%, Indication = 29.6%
Garedow and Tesfaye [50]	Empiric 60.4%, Definitive 23.2%, Prophylaxis 16.4%	19.2	Dose = 19.55%, Duration = 18.8%, Frequency = 22.41%, Unnecessary use of antimicrobials (Indication) = 19.4%
Gebremichael et al. [59]	Empiric 84.0%, Definitive 16.0%	29.2	Disease drug interaction = 3.7%, Dose = 7.5%, Drug-Drug interaction = 9.1%, Duration = 12.2%, Frequency = 11.3%, Indication = 3.7%, Ineffectiveness = 12.3%, Expensive alternativeness = 29.3%, Unnecessary duplication = 10.8%
Gebretekle et al. [60]	Not specified	66.9	Not reported
Tessema et al. [43]	Not specified	Not reported	Indication = 18.77%
Firomsa et al. [48]	Not specified	71.51	Adverse drug events = 16.81%, Ineffective drug therapy = 20.42%, Need additional drug therapy = 22.77%, Non-adherence = 18.94%, Unnecessary use of antimicrobials (Indication) = 21.06%
Gidey et al. [62]	Empiric 67.7%, Prophylaxis 32.3%	53.9	Adverse drug reaction = 0.6%, Dose too high = 13.6%, Dose too low = 19.8%, Ineffective antimicrobial therapy = 12.4%, Need additional antimicrobials = 21.5%, Non-adherence = 3.4%, Unnecessary use of antimicrobials (Indication) = 29.9%
Niriayo et al. [68]	Not specified	61.4	Adverse drug reaction = 2.6%, Dose too high = 14.1%, Dose too low = 8.9%, Ineffective drug therapy = 9.8%, Need additional drug therapy = 32.3%, Non-adherence = 9.4%, Unnecessary use of antimicrobials (Indication) = 14.1%
Yadesa et al. [55]	Not specified	75.7	Adverse drug reactions = 6.8%, Dose too high = 11.2%, Dose too low = 23.3%, Ineffective antimicrobial = 6.8%, Need additional antimicrobial = 23.3%, Unnecessary use of antimicrobials (Indication) = 21.8%, Non-adherence = 14.0%
Tefera et al. [6]	Empiric 58.8%, Prophylaxis 41.2%	69.3	Adverse drug reaction = 2.3%, Choice (Ineffective) = 4.6%, Dose too high = 20.7%, Dose too low = 32.9%, Need additional antibiotic = 4.9%, Unnecessary use of antimicrobials (Indication) = 17.0%, Non-adherence = 5.5%, Unclassified problems = 12.1%

^*^KAH Kahsay Abera Hospital, *MGH* Mearg General Hospital, *ZGH* Zewditu General Hospital, *HGH* Hayat General Hospital

### Factors associated with inappropriate prescribing

Nineteen studies [6, 16, 31–33, 41, 47, 48, 55, 57, 58, 62, 65, 68–70, 74, 77, 78] used statistical analyses, primarily binary logistic regression, to identify factors associated with inappropriate antibiotic prescribing. These factors may be grouped into four categories: patient-related, indication-related, prescriber-related, and diagnostic-related (Table 3).

**Table 3:** Factors associated with inappropriateness of antibiotic prescribing

Study	Factors associated with inappropriate prescribing
Adere et al. [57]	Length of hospital stays (AOR) = 2.97, 95% CI 1.06–8.32; *p* = 0.04, Comorbidities (AOR) = 3.10, 95% CI 1.12–8.15, *p* = 0.02
Afework et al. [58]	Gastrointestinal and Hepato-Pancreato-Biliary surgery (AOR) = 7.89, 95% CI 3.88–20.715.62, *p* = 0.0001
Alekaw et al. [31]	Rural residents (AOR) = 0.574, 95% CI = 0.333– 0.989, *p* = 0.046, Aged < 1 month (AOR) = 4.933, 95% CI = 1.577–15.428, *p* = 0.06 and Age 1 month to 1 year (AOR) = 3.18, 95% CI = 1.166–11.225, *p* = 0.026, Patients whose parents were negligent (AOR) = 6.978, 95% CI 1.872–20.017, *p* = 0.04
Anteneh et al. [32]	Male sex (AOR) = 5.99, 95% CI 2.00–7.98, Patient employed (AOR) = 7.29 95% CI 1.34–9.58, Antibiotics administer after blood culture (AOR) = 2.74 95% CI 1.09–8.37, Antibiotics administer after cerebrospinal fluid culture (AOR) = 5.82, 95% CI 1.84–5.63, Patients believe that the prescribed antibiotics prevent disease complication(s) (AOR) = 4.21 95% CI 1.33–7.35
Ayele et al. [33]	Empiric treatment with ceftriaxone (AOR) = 22.57, 95% CI 4.66–41.47, *p* = 0.001, Co-administered drugs (AOR) = 4.12, 95% CI 1.62–8.05, *p* = 0.002
Firomsa et al. [48]	Place of residence (AOR) = 2.550, 95 CI% 1.238–5.253, *p* = 0.011, Length of hospital stay > 7 days (AOR) = 9.785, 95 CI% 4.668–20.511, *p* = 0.001, Polypharmacy (AOR) = 3.229, 95 CI%: 1.433–7.278, *p* = 0.005
Debela et al. [16]	Positive culture finding (AOR) = 0.32, 95% CI 0.13–0.81, *p* = 0.016, Administered metronidazole (AOR) = 0.25, 95% CI 0.13–0.49, *p* = 0.001, Administered vancomycin (AOR) = 2.93, 95% CI 1.57–5.48, *p* = 0.001
Jambo et al. [78]	Age > 5 years (AOR) = 1.71, CI 95% 1.00–2.94, Age 6 and 14 years (AOR) = 3.14, CI 95% 1.64–6.00, Age < 65 years (AOR = 2.97, CI 95% 1.07–2.66, Comorbidities (AOR) = 1.74, CI 95% 1.10–2.72, Prescribers’ qualification level (AOR) = 1.80, CI 95% 1.14–2.84
Mehari [65]	Empiric (AOR) = 0.069, 95% CI 0.014–0.35 and prophylaxis treatment (AOR) = 0.002, 95% CI 0.001–0.024
Mulat [41]	Availability of first line prophylactic antibiotics (AOR) = 16.834, 95% CI7.687–36.865, *p* = 0.001, Patients after prostatectomy received prophylactic antibiotics (AOR) = 4.115, 95% CI 1.404–12.048, *p* = 0.010, Patients after thyroidectomy received surgical antimicrobial prophylaxis (AOR) = 23.255, 95% CI 2.967–71.428, *p* = 0.001, Patients after hernia repair (AOR) = 48.64, 95% CI 10.6–222.1, *p* = 0.000
Shimels and Fenta [69]	Length of hospital stays (2–7 days) (AOR) = 0.582, 95% CI 0.349- 0.969, Free of charge medical service (AOR) = 0.129, 95% CI 0.047–0.351, Admitted to gynaecology/obstetrics ward (AOR) = 3.228, 95% CI 1.590–6.555, medical ward (AOR) = 3.085, 95% CI 1.436–6.624, paediatrics ward (AOR) = 5.205, 95% CI 1.520–17.822 compared to surgical ward, Member of either the federal or regional police (AOR) = 7, 95% CI 1.973- 24.833
Shimels et al. [77]	Hospital stay < 2 weeks in both the public and the private hospital (AOR) = 6.00; 95% CI 3.40–10.80), (AOR) = 3.30; 95% CI 1.00–9.12
Sileshi et al. [70]	Empiric treatment (AOR) = 36.98, 95% CI 3.884:352.072, *p* = 0.002
Yehualaw et al. [74]	Advanced age of children, children aged between 6 to 10 years (AOR) = 3:225, CI 1:080 − 9:630, P = 036 and 11–18 years (AOR) = 18:691, CI 5:156 − 67:756; P = 0.000 compared to < 1 month
Firomsa et al. [48]	Polypharmacy (AOR) = 2.505, 95% CI 1.863–11.13, *p* = 0.010, Length of hospital stay (⩾7 days) (AOR) = 4.396, 95% CI 1.964–7.310, *p* = 0.037, Comorbidities (AOR) = 2.107, 95% CI 1.185–4.158, *p* = 0.016
Gidey et al. [62]	Length of stay hospital stays (> 1 week) (AOR) = 1.88, 95% CI 1.08–3.26, *p* = 0.025, Comorbidities (AOR) = 1.84, 95% CI 1.04–3.27, *p* = 0.036
Niriayo et al. [68]	Length of hospital stays (> 2 weeks) (AOR) = 3.57, 95% CI 1.91–6.70, *p* = 0.0001, Polypharmacy > 5 drugs (AOR) = 2.08, 95% CI 1.103–3.94, *p* = 0.024, Prescribers’ qualification level (general practitioners (AOR) = 10.27, 95% CI 4.13–25.58, *p* = 0.000 and surgical residents (AOR) = 2.28, 95% CI 1.12–4.63, *p* = 0.023
Yadesa et al. [55]	Coverage of the infectious medical condition in the national guidelines (AOR) = 4.888 95% CI 1.083–22.069, *p* = 0.039, Length of hospital stay (> 3 weeks) (AOR) = 3.099, 95% CI 0.826–11.621 *p* = 0.0094
Tefera et al. [6]	Receiving antibiotics for surgical prophylaxis (AOR) = 6.834, 95% CI 3.025 15.439, *p* = 0.0001 and for both prophylaxis and therapy (AOR) = 8.211, 95% CI 2.215–30.44, *p* = 0.0001, Comorbidities, patients with cardiothoracic conditions have lower odds than those with GIT conditions (AOR) = 0.040, 95% CI 0.003–0.571, *p* = 0.018, CDC wound class I (AOR) = 14.939, 95% CI 1.646–135.56, *p* = 0.016 and II (AOR) = 33.555, 95% CI 3.726–302.209, *p* = 0.002 had higher risks compared to patients without wounds, Polypharmacy (AOR) = 3.343, 95% CI 1.224–9.133, *p* = 0.019, > 3 antibiotic exposures (AOR) = 4.838, 95% CI 1.586–14.752, *p* = 0.006 compared to < 2 antibiotics

*Patient-related factors* were most frequently reported contributors to inappropriate prescribing (n = 14). Increased length of hospital stay was frequently examined as a patient characteristic. Seven studies [46, 48, 55, 57, 62, 68, 77] found higher odds of inappropriate prescribing among patients admitted for more than one to three weeks (AORs 1.88–9.79). Comorbidities were identified in five studies [6, 48, 57, 62, 78], with AORs ranging from 1.74 to 3.10. Four of these studies did not specify the type of comorbidity, but one reported that patients with cardiothoracic conditions were less likely to receive inappropriate prescriptions than those with gastrointestinal conditions (AOR, 0.040) [6]. Polypharmacy was reported in four studies. All demonstrated increased odds of inappropriate prescribing (AOR, 3.229 [47], 2.505 [48], 2.08 [68], and 3.343 [6]. Demographic factors were examined in three studies, though findings were mixed. One study found higher odds among younger age groups (< 1 month: AOR 4.93,1–12 months: AOR 3.18) compared with children aged 10—14 years [31], whereas another reported the reverse pattern with increased odds among older children (6–10 years: AOR 3.23,11–18 years: AOR 18.69) relative to infants < 1 month [74]. A third study found male sex (AOR 5.99) and employment status (AOR 7.29) were associated with appropriate prescribing [32]. Rural residence was linked to higher odds of inappropriate antibiotic use compared with urban settings (AOR 0.57) in one study [31].

*Indication-related factors* were described in five studies [6, 33, 41, 65, 70]. Across these prescribing that was empiric or prophylactic was consistently more likely to be inappropriate compared to culture-guided therapy. Empiric prescribing was associated with markedly increased odds of inappropriate use (AOR, 22.57 [33] and 36.98 [70] while prophylactic prescribing also carried elevated risk (AOR 4.12 [41] and 6.83 [6].

*Prescriber-related factors* were identified in two studies. One found that prescriptions written by medical interns were more likely to be inappropriate compared to specialists (AOR, 1.80 [78]. Another reported increased odds among general practitioners (AOR, 10.27) and surgical residents (AOR, 2.28) compared with surgical specialists [68].

*Diagnostic-related factors* were also described, although the findings were contrasting. One study shows that obtaining cultures results before initiating antibiotics was associated with more appropriate prescribing, whether through blood culture (AOR, 2.74) or cerebrospinal fluid culture (AOR, 5.82) [32]. Another study, however, found that having a positive culture result was linked with inappropriate prescribing [16].

## Discussion

This review provides the first national overview of inappropriate antibiotic prescribing in Ethiopian hospitals, synthesising findings from 53 studies involving more than 17,000 inpatients. Despite a national AMS program introduced in 2011 [80] and Antimicrobial Resistance Prevention and Containment Strategy launched in 2022 [22] inappropriate antibiotic prescribing remains high. The median prevalence was 56.8% per patient and 40.7% per prescription, with frequent deviations from guideline recommendations related to indication, dose, dosing frequency and duration of therapy. Patient-related factors, including comorbidity and polypharmacy, were frequently associated with increased odds of inappropriate antibiotic prescribing.

Median prevalence estimates were 56.8% per patient and 40.7% per prescription, both exceeding recent global estimates of 30% [2] and aligning with patterns seen across LMICs. A global review reported inpatient antibiotic use at 67.5% in low-income settings compared to 37.4% in high-income settings [81], while another LMIC review found inappropriate prescribing ranging from 7.9% to 100%, with more than half of the studies reporting rates above 50% [82]. The wide variation reported across Ethiopian studies should be interpreted considering substantial heterogeneity in how inappropriate prescribing was defined and measured. While some studies assessed prescribing based on parameters such as indication, dose, dosing frequency, and duration of therapy, others incorporated additional variables. These additional parameters were inconsistently applied across studies, leading to differences in the underlying construct of inappropriate prescribing and limiting direct comparability of prevalence estimates. The interpretation of findings should consider the methodological limitations of the included studies, particularly inadequate control for confounding and unclear sampling methods.

These findings are consistent with international evidence highlighting substantial differences between high- and low-resource settings. For example, only 10.8% of prescriptions in Switzerland were issued without a valid indication [83], compared to 37% in Nigeria [84]. Similarly, while 4.7% of prescription in Australia involved an inappropriate duration [85], 67.0% of surgical patients in Tanzania received antibiotics for too long [86]. Prophylactic use was also a major driver for inappropriate antibiotic prescribing in our review (median, 27.0%), a rate comparable to Kenya (24.5%) [87].

These patterns suggest that although the types of inappropriate prescribing are similar across settings, the review also shows both high and highly variable prevalence, ranging from 10.2 to 91.4% across regions, reflects weaker implementation of treatment guidelines [19]. Culture- and sensitivity-guided prescribing, although recommended in the Ethiopian AMR Strategy [22], was rarely reported. Limited diagnostic capacity, inconsistent guideline use, and shortages of AMS-trained staff contribute to heavy reliance on empiric therapy [14], while unnecessary, prolonged prophylaxis, particularly in surgical wards, remains widespread [58, 88]. The frequent reporting of ceftriaxone use across studies underscores this issue: it is most commonly chosen for empiric and prophylactic therapy due to its broad spectrum, perceived safety, affordability, and availability [89]. These patterns indicate that prescribing without diagnostic confirmation continues to drive inappropriate use, reinforcing the importance of timely review and de-escalation of empiric therapy once test results become available [90]. Without such support, unnecessary exposure to broad spectrum agents accelerates the development of AMR [91]. These findings highlight the potential role of pharmacists in strengthening AMS programmes, which have been shown to improve appropriate antibiotic prescribing and clinical outcomes, including reduced mortality and length of hospital stay [28, 92]. In this context, key targets for intervention include optimising antibiotic indication, dosing, and duration, as well as reviewing empiric therapy, de-escalation, and intravenous-to-oral switching supported by pharmacist-led audit and feedback.

This review has some limitations. Considerable heterogeneity in study methods, including differing denominators, guidelines, antibiotics assessed, and patient populations, prevented meta-analysis, requiring narrative synthesis. The use of median values to summarise prevalence estimates reflects this variability and the lack of comparability across studies. Most studies were cross-sectional and single-centre, and many relied on retrospective chart reviews, often with incomplete documentation. Nonetheless, the consistency of findings highlights the scale of the problem. Although we included grey literature such as theses where available, some relevant studies, particularly from local or non-indexed Ethiopian sources, may not have been identified. Future work should use systemic, multi-site designs that assess appropriateness more systematically and explore prescriber perspectives and institutional barriers. Such evidence is vital to inform effective and sustainable AMS strategies in Ethiopia and other LMICs.

## Conclusion

This review suggests that inappropriate antibiotic prescribing is common in Ethiopian hospitals, most commonly involving errors in indication, dose, dosing frequency, and duration. The high prevalence of empiric and prophylactic prescribing may reflect limited diagnostic capacity, while the frequent use of cephalosporins, particularly ceftriaxone, signals accessibility but may raise concerns about resistance and restricted therapeutic options. Together, these findings underscore the need for strengthening of AMS interventions, including routine prescribing audits, strengthened diagnostic services, and adherence to treatment guidelines to support appropriate antibiotic use in Ethiopia.

## Supplementary Information

Below is the link to the electronic supplementary material.

Supplementary file1 (DOCX 29 KB)

Supplementary file2 (DOCX 111 KB)

Supplementary file3 (XLSX 73 KB)

## Data Availability

Data are available from the corresponding author upon request.
