# Supplementary material for: Inappropriate antibiotic prescribing in Ethiopian hospitals: a systematic review
Source: Int J Clin Pharm. 2026 May 12;48(4):1228–45. doi: 10.1007/s11096-026-02157-5 (PMC13369434; doi:10.1007/s11096-026-02157-5)
Supplement: Supplementary file 1 — Supplementary file1 (DOCX 29 KB) [file 11096_2026_2157_MOESM1_ESM.docx]

**Table 1: Search strategy overview for all databases**

| Database | Number of articles |
| --- | --- |
| Medline (Ovid) | 95 |
| Embase (Ovid) | 158 |
| Scopus | 118 |
| Web of Science | 73 |

**Medline (Ovid)= 95**

| 1 | exp Anti-Bacterial Agents/ | 864,494 |
| --- | --- | --- |
| 2 | Antibiotic*.mp. [mp=title, book title, abstract, original title, name of substance word, subject heading word, floating sub-heading word, keyword heading word, organism supplementary concept word, protocol supplementary concept word, rare disease supplementary concept word, unique identifier, synonyms, population supplementary concept word, anatomy supplementary concept word] | 511,969 |
| 3 | Antibacterial*.mp. [mp=title, book title, abstract, original title, name of substance word, subject heading word, floating sub-heading word, keyword heading word, organism supplementary concept word, protocol supplementary concept word, rare disease supplementary concept word, unique identifier, synonyms, population supplementary concept word, anatomy supplementary concept word] | 128,963 |
| 4 | Antimicrobial*.mp. [mp=title, book title, abstract, original title, name of substance word, subject heading word, floating sub-heading word, keyword heading word, organism supplementary concept word, protocol supplementary concept word, rare disease supplementary concept word, unique identifier, synonyms, population supplementary concept word, anatomy supplementary concept word] | 272,446 |
| 5 | Antiinfective*.mp. [mp=title, book title, abstract, original title, name of substance word, subject heading word, floating sub-heading word, keyword heading word, organism supplementary concept word, protocol supplementary concept word, rare disease supplementary concept word, unique identifier, synonyms, population supplementary concept word, anatomy supplementary concept word] | 637 |
| 6 | Anti-infective.mp. [mp=title, book title, abstract, original title, name of substance word, subject heading word, floating sub-heading word, keyword heading word, organism supplementary concept word, protocol supplementary concept word, rare disease supplementary concept word, unique identifier, synonyms, population supplementary concept word, anatomy supplementary concept word] | 93,441 |
| 7 | 1 or 2 or 3 or 4 or 5 or 6 | 1,296,822 |
| 8 | exp Inappropriate Prescribing/ | 5,105 |
| 9 | Inappropriate*.mp. [mp=title, book title, abstract, original title, name of substance word, subject heading word, floating sub-heading word, keyword heading word, organism supplementary concept word, protocol supplementary concept word, rare disease supplementary concept word, unique identifier, synonyms, population supplementary concept word, anatomy supplementary concept word] | 90,773 |
| 10 | Unnecessary.mp. [mp=title, book title, abstract, original title, name of substance word, subject heading word, floating sub-heading word, keyword heading word, organism supplementary concept word, protocol supplementary concept word, rare disease supplementary concept word, unique identifier, synonyms, population supplementary concept word, anatomy supplementary concept word] | 77,342 |
| 11 | Misuse*.mp. [mp=title, book title, abstract, original title, name of substance word, subject heading word, floating sub-heading word, keyword heading word, organism supplementary concept word, protocol supplementary concept word, rare disease supplementary concept word, unique identifier, synonyms, population supplementary concept word, anatomy supplementary concept word] | 35,564 |
| 12 | Over prescribe*.mp. [mp=title, book title, abstract, original title, name of substance word, subject heading word, floating sub-heading word, keyword heading word, organism supplementary concept word, protocol supplementary concept word, rare disease supplementary concept word, unique identifier, synonyms, population supplementary concept word, anatomy supplementary concept word] | 167 |
| 13 | Over-prescribe*.mp. [mp=title, book title, abstract, original title, name of substance word, subject heading word, floating sub-heading word, keyword heading word, organism supplementary concept word, protocol supplementary concept word, rare disease supplementary concept word, unique identifier, synonyms, population supplementary concept word, anatomy supplementary concept word] | 167 |
| 14 | Overutiliz*.mp. [mp=title, book title, abstract, original title, name of substance word, subject heading word, floating sub-heading word, keyword heading word, organism supplementary concept word, protocol supplementary concept word, rare disease supplementary concept word, unique identifier, synonyms, population supplementary concept word, anatomy supplementary concept word] | 1,178 |
| 15 | Overutilis*.mp. [mp=title, book title, abstract, original title, name of substance word, subject heading word, floating sub-heading word, keyword heading word, organism supplementary concept word, protocol supplementary concept word, rare disease supplementary concept word, unique identifier, synonyms, population supplementary concept word, anatomy supplementary concept word] | 74 |
| 16 | Over-utilis*.mp. [mp=title, book title, abstract, original title, name of substance word, subject heading word, floating sub-heading word, keyword heading word, organism supplementary concept word, protocol supplementary concept word, rare disease supplementary concept word, unique identifier, synonyms, population supplementary concept word, anatomy supplementary concept word] | 59 |
| 17 | 8 or 9 or 10 or 11 or 12 or 13 or 14 or 15 or 16 | 198,706 |
| 18 | exp Prescriptions/ | 43,674 |
| 19 | Drug*.mp. [mp=title, book title, abstract, original title, name of substance word, subject heading word, floating sub-heading word, keyword heading word, organism supplementary concept word, protocol supplementary concept word, rare disease supplementary concept word, unique identifier, synonyms, population supplementary concept word, anatomy supplementary concept word] | 7,057,774 |
| 20 | Prescribe*.mp. [mp=title, book title, abstract, original title, name of substance word, subject heading word, floating sub-heading word, keyword heading word, organism supplementary concept word, protocol supplementary concept word, rare disease supplementary concept word, unique identifier, synonyms, population supplementary concept word, anatomy supplementary concept word] | 166,122 |
| 21 | Prescription.mp. [mp=title, book title, abstract, original title, name of substance word, subject heading word, floating sub-heading word, keyword heading word, organism supplementary concept word, protocol supplementary concept word, rare disease supplementary concept word, unique identifier, synonyms, population supplementary concept word, anatomy supplementary concept word] | 123,152 |
| 22 | Over prescribe*.mp. [mp=title, book title, abstract, original title, name of substance word, subject heading word, floating sub-heading word, keyword heading word, organism supplementary concept word, protocol supplementary concept word, rare disease supplementary concept word, unique identifier, synonyms, population supplementary concept word, anatomy supplementary concept word] | 167 |
| 23 | Over-prescribe*.mp. [mp=title, book title, abstract, original title, name of substance word, subject heading word, floating sub-heading word, keyword heading word, organism supplementary concept word, protocol supplementary concept word, rare disease supplementary concept word, unique identifier, synonyms, population supplementary concept word, anatomy supplementary concept word] | 167 |
| 24 | Overutiliz*.mp. [mp=title, book title, abstract, original title, name of substance word, subject heading word, floating sub-heading word, keyword heading word, organism supplementary concept word, protocol supplementary concept word, rare disease supplementary concept word, unique identifier, synonyms, population supplementary concept word, anatomy supplementary concept word] | 1,178 |
| 25 | Overutilis*.mp. [mp=title, book title, abstract, original title, name of substance word, subject heading word, floating sub-heading word, keyword heading word, organism supplementary concept word, protocol supplementary concept word, rare disease supplementary concept word, unique identifier, synonyms, population supplementary concept word, anatomy supplementary concept word] | 74 |
| 26 | Ceftriaxone.mp. [mp=title, book title, abstract, original title, name of substance word, subject heading word, floating sub-heading word, keyword heading word, organism supplementary concept word, protocol supplementary concept word, rare disease supplementary concept word, unique identifier, synonyms, population supplementary concept word, anatomy supplementary concept word] | 17,046 |
| 27 | Cefepime.mp. [mp=title, book title, abstract, original title, name of substance word, subject heading word, floating sub-heading word, keyword heading word, organism supplementary concept word, protocol supplementary concept word, rare disease supplementary concept word, unique identifier, synonyms, population supplementary concept word, anatomy supplementary concept word] | 5,131 |
| 28 | Beta lactam*.mp. [mp=title, book title, abstract, original title, name of substance word, subject heading word, floating sub-heading word, keyword heading word, organism supplementary concept word, protocol supplementary concept word, rare disease supplementary concept word, unique identifier, synonyms, population supplementary concept word, anatomy supplementary concept word] | 67,768 |
| 29 | Ciprofloxacin.mp. [mp=title, book title, abstract, original title, name of substance word, subject heading word, floating sub-heading word, keyword heading word, organism supplementary concept word, protocol supplementary concept word, rare disease supplementary concept word, unique identifier, synonyms, population supplementary concept word, anatomy supplementary concept word] | 37,528 |
| 30 | Metronidazole.mp. [mp=title, book title, abstract, original title, name of substance word, subject heading word, floating sub-heading word, keyword heading word, organism supplementary concept word, protocol supplementary concept word, rare disease supplementary concept word, unique identifier, synonyms, population supplementary concept word, anatomy supplementary concept word] | 23,619 |
| 31 | 18 or 19 or 20 or 21 or 22 or 23 or 24 or 25 or 26 or 27 or 28 or 29 or 30 | 7,202,202 |
| 32 | exp Hospitals/ | 333,807 |
| 33 | Hospital*.mp. [mp=title, book title, abstract, original title, name of substance word, subject heading word, floating sub-heading word, keyword heading word, organism supplementary concept word, protocol supplementary concept word, rare disease supplementary concept word, unique identifier, synonyms, population supplementary concept word, anatomy supplementary concept word] | 2,160,516 |
| 34 | Hospital setting*.mp. [mp=title, book title, abstract, original title, name of substance word, subject heading word, floating sub-heading word, keyword heading word, organism supplementary concept word, protocol supplementary concept word, rare disease supplementary concept word, unique identifier, synonyms, population supplementary concept word, anatomy supplementary concept word] | 23,444 |
| 35 | Hospital-setting*.mp. [mp=title, book title, abstract, original title, name of substance word, subject heading word, floating sub-heading word, keyword heading word, organism supplementary concept word, protocol supplementary concept word, rare disease supplementary concept word, unique identifier, synonyms, population supplementary concept word, anatomy supplementary concept word] | 23,444 |
| 36 | Inpatient*.mp. [mp=title, book title, abstract, original title, name of substance word, subject heading word, floating sub-heading word, keyword heading word, organism supplementary concept word, protocol supplementary concept word, rare disease supplementary concept word, unique identifier, synonyms, population supplementary concept word, anatomy supplementary concept word] | 167,790 |
| 37 | Clinical ward*.mp. [mp=title, book title, abstract, original title, name of substance word, subject heading word, floating sub-heading word, keyword heading word, organism supplementary concept word, protocol supplementary concept word, rare disease supplementary concept word, unique identifier, synonyms, population supplementary concept word, anatomy supplementary concept word] | 363 |
| 38 | Critical Care.mp. [mp=title, book title, abstract, original title, name of substance word, subject heading word, floating sub-heading word, keyword heading word, organism supplementary concept word, protocol supplementary concept word, rare disease supplementary concept word, unique identifier, synonyms, population supplementary concept word, anatomy supplementary concept word] | 97,555 |
| 39 | Paediatric*.mp. [mp=title, book title, abstract, original title, name of substance word, subject heading word, floating sub-heading word, keyword heading word, organism supplementary concept word, protocol supplementary concept word, rare disease supplementary concept word, unique identifier, synonyms, population supplementary concept word, anatomy supplementary concept word] | 96,753 |
| 40 | Adult*.mp. [mp=title, book title, abstract, original title, name of substance word, subject heading word, floating sub-heading word, keyword heading word, organism supplementary concept word, protocol supplementary concept word, rare disease supplementary concept word, unique identifier, synonyms, population supplementary concept word, anatomy supplementary concept word] | 6,902,169 |
| 41 | Intensive care unit.mp. [mp=title, book title, abstract, original title, name of substance word, subject heading word, floating sub-heading word, keyword heading word, organism supplementary concept word, protocol supplementary concept word, rare disease supplementary concept word, unique identifier, synonyms, population supplementary concept word, anatomy supplementary concept word] | 151,303 |
| 42 | Surgical.mp. [mp=title, book title, abstract, original title, name of substance word, subject heading word, floating sub-heading word, keyword heading word, organism supplementary concept word, protocol supplementary concept word, rare disease supplementary concept word, unique identifier, synonyms, population supplementary concept word, anatomy supplementary concept word] | 1,716,423 |
| 43 | Emergency.mp. [mp=title, book title, abstract, original title, name of substance word, subject heading word, floating sub-heading word, keyword heading word, organism supplementary concept word, protocol supplementary concept word, rare disease supplementary concept word, unique identifier, synonyms, population supplementary concept word, anatomy supplementary concept word] | 442,263 |
| 44 | Orthopaedic.mp. [mp=title, book title, abstract, original title, name of substance word, subject heading word, floating sub-heading word, keyword heading word, organism supplementary concept word, protocol supplementary concept word, rare disease supplementary concept word, unique identifier, synonyms, population supplementary concept word, anatomy supplementary concept word] | 51,693 |
| 45 | Gynaecology.mp. [mp=title, book title, abstract, original title, name of substance word, subject heading word, floating sub-heading word, keyword heading word, organism supplementary concept word, protocol supplementary concept word, rare disease supplementary concept word, unique identifier, synonyms, population supplementary concept word, anatomy supplementary concept word] | 10,191 |
| 46 | Obstetric.mp. [mp=title, book title, abstract, original title, name of substance word, subject heading word, floating sub-heading word, keyword heading word, organism supplementary concept word, protocol supplementary concept word, rare disease supplementary concept word, unique identifier, synonyms, population supplementary concept word, anatomy supplementary concept word] | 138,566 |
| 47 | Outpatient.mp. [mp=title, book title, abstract, original title, name of substance word, subject heading word, floating sub-heading word, keyword heading word, organism supplementary concept word, protocol supplementary concept word, rare disease supplementary concept word, unique identifier, synonyms, population supplementary concept word, anatomy supplementary concept word] | 195,939 |
| 48 | Oncology.mp. [mp=title, book title, abstract, original title, name of substance word, subject heading word, floating sub-heading word, keyword heading word, organism supplementary concept word, protocol supplementary concept word, rare disease supplementary concept word, unique identifier, synonyms, population supplementary concept word, anatomy supplementary concept word] | 184,488 |
| 49 | Ward*.mp. [mp=title, book title, abstract, original title, name of substance word, subject heading word, floating sub-heading word, keyword heading word, organism supplementary concept word, protocol supplementary concept word, rare disease supplementary concept word, unique identifier, synonyms, population supplementary concept word, anatomy supplementary concept word] | 80,249 |
| 50 | 32 or 33 or 34 or 35 or 36 or 37 or 38 or 39 or 40 or 41 or 42 or 43 or 44 or 45 or 46 or 47 or 48 or 49 | 10,012,825 |
| 51 | exp Ethiopia/ | 23,392 |
| 52 | Ethiopia*.mp. [mp=title, book title, abstract, original title, name of substance word, subject heading word, floating sub-heading word, keyword heading word, organism supplementary concept word, protocol supplementary concept word, rare disease supplementary concept word, unique identifier, synonyms, population supplementary concept word, anatomy supplementary concept word] | 39,190 |
| 53 | 51 or 52 | 39,190 |
| 54 | 7 and 17 and 31 and 50 and 53 | 95 |

**Embase (Ovid)= 158**

| 1 | exp antibiotic agent/ | 2,016,150 |
| --- | --- | --- |
| 2 | Antibiotic*.mp. [mp=title, abstract, heading word, drug trade name, original title, device manufacturer, drug manufacturer, device trade name, keyword heading word, floating subheading word, candidate term word] | 1,036,106 |
| 3 | Antibacterial*.mp. [mp=title, abstract, heading word, drug trade name, original title, device manufacturer, drug manufacturer, device trade name, keyword heading word, floating subheading word, candidate term word] | 204,459 |
| 4 | Antimicrobial*.mp. [mp=title, abstract, heading word, drug trade name, original title, device manufacturer, drug manufacturer, device trade name, keyword heading word, floating subheading word, candidate term word] | 375,308 |
| 5 | Antiinfective*.mp. [mp=title, abstract, heading word, drug trade name, original title, device manufacturer, drug manufacturer, device trade name, keyword heading word, floating subheading word, candidate term word] | 270,755 |
| 6 | Anti-infective.mp. [mp=title, abstract, heading word, drug trade name, original title, device manufacturer, drug manufacturer, device trade name, keyword heading word, floating subheading word, candidate term word] | 9,464 |
| 7 | 1 or 2 or 3 or 4 or 5 or 6 | 2,505,502 |
| 8 | exp potentially inappropriate medication/ | 3,339 |
| 9 | Inappropriate*.mp. [mp=title, abstract, heading word, drug trade name, original title, device manufacturer, drug manufacturer, device trade name, keyword heading word, floating subheading word, candidate term word] | 134,453 |
| 10 | Unnecessary.mp. [mp=title, abstract, heading word, drug trade name, original title, device manufacturer, drug manufacturer, device trade name, keyword heading word, floating subheading word, candidate term word] | 113,366 |
| 11 | Misuse*.mp. [mp=title, abstract, heading word, drug trade name, original title, device manufacturer, drug manufacturer, device trade name, keyword heading word, floating subheading word, candidate term word] | 45,342 |
| 12 | Over prescribe*.mp. [mp=title, abstract, heading word, drug trade name, original title, device manufacturer, drug manufacturer, device trade name, keyword heading word, floating subheading word, candidate term word] | 289 |
| 13 | Over-prescribe*.mp. [mp=title, abstract, heading word, drug trade name, original title, device manufacturer, drug manufacturer, device trade name, keyword heading word, floating subheading word, candidate term word] | 289 |
| 14 | Overutiliz*.mp. [mp=title, abstract, heading word, drug trade name, original title, device manufacturer, drug manufacturer, device trade name, keyword heading word, floating subheading word, candidate term word] | 1,847 |
| 15 | Overutilis*.mp. [mp=title, abstract, heading word, drug trade name, original title, device manufacturer, drug manufacturer, device trade name, keyword heading word, floating subheading word, candidate term word] | 95 |
| 16 | Over-utilis*.mp. [mp=title, abstract, heading word, drug trade name, original title, device manufacturer, drug manufacturer, device trade name, keyword heading word, floating subheading word, candidate term word] | 115 |
| 17 | 8 or 9 or 10 or 11 or 12 or 13 or 14 or 15 or 16 | 287,087 |
| 18 | exp prescription drug misuse/ | 2,957 |
| 19 | Drug*.mp. [mp=title, abstract, heading word, drug trade name, original title, device manufacturer, drug manufacturer, device trade name, keyword heading word, floating subheading word, candidate term word] | 14,174,510 |
| 20 | Prescribe*.mp. [mp=title, abstract, heading word, drug trade name, original title, device manufacturer, drug manufacturer, device trade name, keyword heading word, floating subheading word, candidate term word] | 288,218 |
| 21 | Prescription.mp. [mp=title, abstract, heading word, drug trade name, original title, device manufacturer, drug manufacturer, device trade name, keyword heading word, floating subheading word, candidate term word] | 366,586 |
| 22 | Over prescribe*.mp. [mp=title, abstract, heading word, drug trade name, original title, device manufacturer, drug manufacturer, device trade name, keyword heading word, floating subheading word, candidate term word] | 289 |
| 23 | Over-prescribe*.mp. [mp=title, abstract, heading word, drug trade name, original title, device manufacturer, drug manufacturer, device trade name, keyword heading word, floating subheading word, candidate term word] | 289 |
| 24 | Overutiliz*.mp. [mp=title, abstract, heading word, drug trade name, original title, device manufacturer, drug manufacturer, device trade name, keyword heading word, floating subheading word, candidate term word] | 1,847 |
| 25 | Overutilis*.mp. [mp=title, abstract, heading word, drug trade name, original title, device manufacturer, drug manufacturer, device trade name, keyword heading word, floating subheading word, candidate term word] | 95 |
| 26 | Ceftriaxone.mp. [mp=title, abstract, heading word, drug trade name, original title, device manufacturer, drug manufacturer, device trade name, keyword heading word, floating subheading word, candidate term word] | 92,125 |
| 27 | Cefepime.mp. [mp=title, abstract, heading word, drug trade name, original title, device manufacturer, drug manufacturer, device trade name, keyword heading word, floating subheading word, candidate term word] | 34,444 |
| 28 | Beta lactam*.mp. [mp=title, abstract, heading word, drug trade name, original title, device manufacturer, drug manufacturer, device trade name, keyword heading word, floating subheading word, candidate term word] | 103,541 |
| 29 | Ciprofloxacin.mp. [mp=title, abstract, heading word, drug trade name, original title, device manufacturer, drug manufacturer, device trade name, keyword heading word, floating subheading word, candidate term word] | 139,428 |
| 30 | Metronidazole.mp. [mp=title, abstract, heading word, drug trade name, original title, device manufacturer, drug manufacturer, device trade name, keyword heading word, floating subheading word, candidate term word] | 90,292 |
| 31 | 18 or 19 or 20 or 21 or 22 or 23 or 24 or 25 or 26 or 27 or 28 or 29 or 30 | 14,402,273 |
| 32 | exp hospital/ | 1,564,840 |
| 33 | Hospital*.mp. [mp=title, abstract, heading word, drug trade name, original title, device manufacturer, drug manufacturer, device trade name, keyword heading word, floating subheading word, candidate term word] | 3,677,254 |
| 34 | Hospital setting*.mp. [mp=title, abstract, heading word, drug trade name, original title, device manufacturer, drug manufacturer, device trade name, keyword heading word, floating subheading word, candidate term word] | 34,991 |
| 35 | Hospital-setting*.mp. [mp=title, abstract, heading word, drug trade name, original title, device manufacturer, drug manufacturer, device trade name, keyword heading word, floating subheading word, candidate term word] | 34,991 |
| 36 | Inpatient*.mp. [mp=title, abstract, heading word, drug trade name, original title, device manufacturer, drug manufacturer, device trade name, keyword heading word, floating subheading word, candidate term word] | 271,553 |
| 37 | Clinical ward*.mp. [mp=title, abstract, heading word, drug trade name, original title, device manufacturer, drug manufacturer, device trade name, keyword heading word, floating subheading word, candidate term word] | 648 |
| 38 | Critical Care.mp. [mp=title, abstract, heading word, drug trade name, original title, device manufacturer, drug manufacturer, device trade name, keyword heading word, floating subheading word, candidate term word] | 84,518 |
| 39 | Paediatric*.mp. [mp=title, abstract, heading word, drug trade name, original title, device manufacturer, drug manufacturer, device trade name, keyword heading word, floating subheading word, candidate term word] | 166,249 |
| 40 | Adult*.mp. [mp=title, abstract, heading word, drug trade name, original title, device manufacturer, drug manufacturer, device trade name, keyword heading word, floating subheading word, candidate term word] | 11,277,518 |
| 41 | Intensive care unit.mp. [mp=title, abstract, heading word, drug trade name, original title, device manufacturer, drug manufacturer, device trade name, keyword heading word, floating subheading word, candidate term word] | 384,103 |
| 42 | Surgical.mp. [mp=title, abstract, heading word, drug trade name, original title, device manufacturer, drug manufacturer, device trade name, keyword heading word, floating subheading word, candidate term word] | 2,218,877 |
| 43 | Emergency.mp. [mp=title, abstract, heading word, drug trade name, original title, device manufacturer, drug manufacturer, device trade name, keyword heading word, floating subheading word, candidate term word] | 769,814 |
| 44 | Orthopaedic.mp. [mp=title, abstract, heading word, drug trade name, original title, device manufacturer, drug manufacturer, device trade name, keyword heading word, floating subheading word, candidate term word] | 72,372 |
| 45 | Gynaecology.mp. [mp=title, abstract, heading word, drug trade name, original title, device manufacturer, drug manufacturer, device trade name, keyword heading word, floating subheading word, candidate term word] | 18,862 |
| 46 | Obstetric.mp. [mp=title, abstract, heading word, drug trade name, original title, device manufacturer, drug manufacturer, device trade name, keyword heading word, floating subheading word, candidate term word] | 155,119 |
| 47 | Outpatient.mp. [mp=title, abstract, heading word, drug trade name, original title, device manufacturer, drug manufacturer, device trade name, keyword heading word, floating subheading word, candidate term word] | 438,877 |
| 48 | Oncology.mp. [mp=title, abstract, heading word, drug trade name, original title, device manufacturer, drug manufacturer, device trade name, keyword heading word, floating subheading word, candidate term word] | 413,321 |
| 49 | Ward*.mp. [mp=title, abstract, heading word, drug trade name, original title, device manufacturer, drug manufacturer, device trade name, keyword heading word, floating subheading word, candidate term word] | 381,803 |
| 50 | 32 or 33 or 34 or 35 or 36 or 37 or 38 or 39 or 40 or 41 or 42 or 43 or 44 or 45 or 46 or 47 or 48 or 49 | 15,071,555 |
| 51 | exp Ethiopia/ | 32,772 |
| 52 | Ethiopia*.mp. [mp=title, abstract, heading word, drug trade name, original title, device manufacturer, drug manufacturer, device trade name, keyword heading word, floating subheading word, candidate term word] | 39,773 |
| 53 | 51 or 52 | 39,773 |
| 54 | 7 and 17 and 31 and 50 and 53 | 158 |

**Scopus= 118**

| ( TITLE-ABS-KEY ( antibiotic* ) OR TITLE-ABS-KEY ( antibacterial* ) OR TITLE-ABS-KEY ( Antimicrobial* ) OR TITLE-ABS-KEY ( Anti-infective ) OR TITLE-ABS-KEY ( Antimicrobial* ) ) AND ( TITLE-ABS-KEY ( Inappropriate* ) OR TITLE-ABS-KEY ( Unnecessary Misuse* ) OR TITLE-ABS-KEY ( Over prescribe* ) OR TITLE-ABS-KEY ( Overutiliz* ) OR TITLE-ABS-KEY ( Overutilis* ) OR TITLE-ABS-KEY ( Over-utilis* ) ) AND ( TITLE-ABS-KEY ( Drug* ) OR TITLE-ABS-KEY ( Prescribe* ) OR TITLE-ABS-KEY ( Prescription Over prescribe* ) OR TITLE-ABS-KEY ( Over-prescribe* ) OR TITLE-ABS-KEY ( Overutilis* ) OR TITLE-ABS-KEY ( Ceftriaxone ) OR TITLE-ABS-KEY ( Cefepime ) OR TITLE-ABS-KEY ( Beta lactam* ) OR TITLE-ABS-KEY ( Ciprofloxacin ) OR TITLE-ABS-KEY ( Metronidazole ) ) AND ( TITLE-ABS-KEY ( Hospital* ) OR TITLE-ABS-KEY ( Hospital setting* ) OR TITLE-ABS-KEY ( Hospital-setting* ) OR TITLE-ABS-KEY ( Inpatient* ) OR TITLE-ABS-KEY ( Clinical ward* ) OR TITLE-ABS-KEY ( Critical Care Paediatric* ) OR TITLE-ABS-KEY ( Adult* ) OR TITLE-ABS-KEY ( Intensive care unit ) OR TITLE-ABS-KEY ( Surgical ) OR TITLE-ABS-KEY ( Emergency ) OR TITLE-ABS-KEY ( Orthopaedic ) OR TITLE-ABS-KEY ( Gynaecology ) OR TITLE-ABS-KEY ( Obstetric ) OR TITLE-ABS-KEY ( Oncology Ward* ) ) AND TITLE-ABS-KEY ( Ethiopia* ) |
| --- |

**Web of Science= 73**

| Antibiotic* OR Antibacterial* OR Antimicrobial* OR Antiinfective* OR Anti-infective (Topic) and Inappropriate* OR Unnecessary OR Misuse* OR Over prescribe* OR Over-prescribe* OR Overutiliz* OR Overutilis* OR Over-utilis* (Topic) and Prescription OR Drug* OR Prescribe* OR Prescription OR Over prescribe* OR Over-prescribe* OR Overutiliz*OR Overutilis* OR Ceftriaxone OR Cefepime OR Beta lactam* OR Ciprofloxacin OR Metronidazole (Topic) and Hospital* OR Hospital setting* OR Hospital-setting* OR Inpatient* OR Clinical ward* OR Critical Care OR Paediatric* OR Adult* OR Intensive care unit OR Surgical OR Emergency OR Orthopaedic OR Gynaecology OR Obstetric OR Outpatient OR Oncology OR Ward* (Topic) and Ethiopia* (Topic) and Preprint Citation Index (Exclude – Database) and Review Article (Exclude – Document Types) and Article (Document Types) and Humans (MeSH Headings) and ETHIOPIA (Countries/Regions) |
| --- |
