# Supplementary material for: Inappropriate antibiotic prescribing in Ethiopian hospitals: a systematic review
Source: Int J Clin Pharm. 2026 May 12;48(4):1228–45. doi: 10.1007/s11096-026-02157-5 (PMC13369434; doi:10.1007/s11096-026-02157-5)
Supplement: Supplementary file 2 — Supplementary file2 (DOCX 111 KB) [file 11096_2026_2157_MOESM2_ESM.docx]

**Table 2A: Critical appraisal results for cross sectional studies in the review**

| **Studies** | **Critical appraisal questions** | | | | | | | |
| --- | --- | --- | --- | --- | --- | --- | --- | --- |
|  | Q1 | Q2 | Q3 | Q4 | Q5 | Q6 | Q7 | Q8 |
| #2. (Shegute et al., 2023) | Y | Y | Y | Y | N | N | Y | Y |
| #3 (Habteweld et al., 2023) | Y | Y | Y | Y | Y | Y | Y | Y |
| #5 (Gidey et al., 2015) | Y | Y | Y | UC | N | N | Y | UC |
| #9 (Gube et al., 2017) | N | Y | N | Y | N | N | Y | Y |
| #12 (Kefale et al., 2020) | Y | Y | Y | Y | Y | Y | Y | UC |
| #13 (Ketema et al., 2019) | UC | Y | Y | UC | N | N | UC | Y |
| #17 (Amare et al., 2021) | UC | Y | Y | Y | N | N | Y | Y |
| #18 (Afework et al., 2023) | Y | Y | UC | Y | Y | Y | Y | Y |
| #19 (Sileshi et al., 2016) | Y | Y | Y | Y | Y | Y | Y | Y |
| #21 (Asefa et al., 2016) | UC | Y | Y | Y | N | N | Y | Y |
| #23 (Adugna et al., 2023) | Y | Y | Y | Y | N | N | Y | Y |
| #27 (Birarra et al., 2022) | Y | Y | Y | Y | N | N | Y | UC |
| #30 (Mohamoud et al., 2016) | Y | Y | Y | Y | N | N | Y | Y |
| #31 (Mulat, 2023) | Y | Y | Y | Y | Y | Y | Y | Y |
| #32 (Moges et al., 2020) | Y | Y | Y | Y | Y | Y | Y | Y |
| #33 (Ayele et al., 2018) | Y | Y | Y | Y | Y | Y | Y | Y |
| #34 (Mengesha & Mohammed, 2025) | Y | Y | Y | N | UC | N | Y | Y |
| #35 (Tassew et al., 2020) | Y | Y | Y | Y | Y | Y | Y | Y |
| #37 (Alekaw et al., 2022) | Y | Y | Y | Y | Y | Y | UC | UC |
| #39 (Firomsa et al., 2019) | Y | Y | Y | Y | Y | N | Y | UC |
| #40 (Fekadu et al., 2017) | Y | Y | Y | Y | N | N | N | UC |
| #41 (Gebremichael et al., 2019) | Y | Y | Y | Y | Y | N | Y | Y |
| #42 (Mama et al., 2020) | N | Y | Y | UC | N | N | Y | Y |
| #43 (Tessema et al., 2014) | N | Y | Y | UC | N | N | Y | UC |
| #46 (Abebe et al., 2012) | N | Y | Y | N | N | N | UC | UC |
| #48 (Muhammed & Nasir, 2020) | N | Y | Y | Y | N | N | Y | Y |
| #49 (Jambo et al., 2023) | Y | Y | Y | Y | Y | Y | Y | Y |
| #51 (Yehualaw et al., 2021) | N | Y | Y | Y | Y | Y | Y | Y |
| #53 (Garedow & Tesfaye, 2022) | Y | Y | Y | Y | Y | Y | Y | UC |
| #54 (Mehari, 2017) | Y | Y | Y | Y | Y | Y | Y | Y |
| #55 (Kebede et al., 2018) | N | Y | Y | Y | N | N | Y | Y |
| #56 (Sewagegn et al., 2017) | N | Y | Y | Y | N | N | Y | Y |
| #57 (Werede et al., 2023) | N | Y | N | UC | N | N | Y | Y |
| #58 (Shimels & Fenta, 2016) | N | Y | Y | UC | Y | Y | Y | Y |
| #59 (Taressa et al., 2021) | N | Y | Y | Y | N | N | Y | UC |
| #60 (Geresu et al., 2018) | N | Y | Y | Y | Y | N | Y | Y |
| #61 (Jifar et al., 2022) | Y | Y | Y | Y | N | N | Y | Y |
| #62 (Ayinalem et al., 2013) | Y | Y | Y | Y | N | N | Y | Y |
| #63 (Bantie, 2014) | N | Y | Y | Y | N | N | UC | UC |
| #64 (Shimels et al., 2015) | Y | Y | Y | Y | Y | Y | Y | Y |
| #47 (Gashaw et al., 2025) | UC | Y | Y | UC | Y | N | Y | Y |

Y= yes, N= no, UC= unclear, NA= not applicable

Q1. Were the criteria for inclusion in the sample clearly defined?

Q2. Were the study subjects and the setting described in detail?

Q3. Was the exposure measured in a valid and reliable way?

Q4. Were objective, standard criteria used for measurement of the condition?

Q5. Were confounding factors identified?

Q6. Were strategies to deal with confounding factors stated?

Q7. Were the outcomes measured in a valid and reliable way?

Q8. Was appropriate statistical analysis used?

**Table 2B: Critical appraisal results for cohort studies in the review**

| **Studies** | **Critical appraisal questions** | | | | | | | | | | |
| --- | --- | --- | --- | --- | --- | --- | --- | --- | --- | --- | --- |
|  | Q1 | Q2 | Q3 | Q4 | Q5 | Q6 | Q7 | Q8 | Q9 | Q10 | Q11 |
| #4 (Gidey et al., 2024) | NA | NA | Y | Y | Y | Y | UC | Y | Y | UC | Y |
| #6 (Wondm et al., 2022) | NA | NA | Y | Y | Y | Y | Y | UC | UC | UC | Y |
| #8 (Niriayo et al., 2023) | NA | NA | Y | Y | Y | Y | Y | Y | Y | UC | Y |
| #15 (Tefera et al., 2019) | NA | NA | Y | Y | Y | Y | Y | Y | Y | N | Y |
| #22 (Belayneh et al., 2018) | NA | NA | Y | UC | N | Y | Y | Y | UC | N | N |
| #24 (Debela et al., 2022) | NA | NA | Y | Y | Y | Y | Y | UC | UC | N | Y |
| #25 (Anteneh et al., 2021) | NA | NA | Y | Y | Y | Y | Y | UC | UC | N | Y |
| #28 (Firomsa, Ginenus, et al., 2021) | NA | NA | Y | Y | Y | Y | Y | N | N | N | Y |
| #29 (Yadesa et al., 2015) | NA | NA | Y | Y | Y | Y | Y | N | UC | N | Y |
| #36 (Adere et al., 2022) | NA | NA | Y | Y | Y | Y | UC | UC | UC | N | Y |
| #52 (Firomsa, Tesfaye, et al., 2021) | NA | NA | Y | Y | Y | Y | Y | UC | UN | N | Y |

Y= yes, N= no, NA= not applicable, UC= unclear

Q1. Were the two groups similar and recruited from the same population?

Q2. Were the exposures measured similarly to assign people to both exposed and unexposed groups?

Q3. Was the exposure measured in a valid and reliable way?

Q4. Were confounding factors identified?

Q5. Were strategies to deal with confounding factors stated?

Q6. Were the groups/participants free of the outcome at the start of the study (or at the moment of exposure)?

Q7. Were the outcomes measured in a valid and reliable way?

Q8. Was the follow up time reported and sufficient to be long enough for outcomes to occur?

Q9. Was follow up complete, and if not, were the reasons to loss to follow up described and explored?

Q10. Were strategies to address incomplete follow up utilized?

Q11. Was appropriate statistical analysis used?

**Table 2C: Critical appraisal results for quasi experimental study design in the review**

| **Studies** | **Critical appraisal questions** | | | | | | | | |
| --- | --- | --- | --- | --- | --- | --- | --- | --- | --- |
|  | Q1 | Q2 | Q3 | Q4 | Q5 | Q6 | Q7 | Q8 | Q9 |
| #45 (Gebretekle. et al., 2020) | Y | N | Y | Y | Y | Y | Y | Y | Y |

Y= yes, N= no

Q1. Is it clear in the study what is the “cause” and what is the “effect” (i.e. there is no confusion about which variable comes first)?

Q2. Was there a control group?

Q3. Were participants included in any comparisons similar?

Q4. Were the participants included in any comparisons receiving similar treatment/care, other than the exposure or intervention of interest?

Q5. Were there multiple measurements of the outcome, both pre and post the intervention/exposure?

Q6. Were the outcomes of participants included in any comparisons measured in the same way?

Q7. Were outcomes measured in a reliable way?

Q8. Was follow-up complete and if not, were differences between groups in terms of their follow-up adequately described and analyzed?

Q9. Was appropriate statistical analysis used?

**References**

Abebe, F. A., Berhe, D. F., Berhe, A. H., Hishe, H. Z., & Akaleweld, M. A. (2012). Drug use evaluation of ceftriaxone: The case of Ayder Referral Hospital, Mekelle, Ethiopia. *International Journal of Pharmaceutical Sciences and Research*, *3(7)*, 2191-2195. <http://www.ijpsr.com/V3I7/43%20Vol.%203,%20Issue%207,%20July%202012,%20RA-1369,%20Paper%2043.pdf>

Adere, A., Edao, A., Tesfaye, M., & Petros, Z. (2022). Antimicrobial use-related drug therapy problems and associated factors among patients in the medical ward of Wachemo University Nigist Eleni Mohammed Memorial Comprehensive Specialized Hospital, Southwest Ethiopia. *SAGE Open Medicine*, *10*, 1-9. <https://doi.org/10.1177/20503121221140228>

Adugna, B. Y., Dlie, Z. Y., Mekonnen, B. A., & Kassaw, A. T. (2023). Evaluation of Vancomycin Utilization in the Medical and Gynecology Wards of Felege Hiwot Comprehensive Specialized Hospital, Northwest Ethiopia. *Journal of Clinical Pharmacy and Therapeutics*, *10*, 1-7. <https://doi.org/10.1155/2023/2335694>

Afework, V., Kejela, S., & Abebe, N. S. (2023). "A breach in the protocol for no good reason": a surgical antimicrobial prophylaxis experience in an Ethiopian academic medical center. *Perioperative Medicine*, *12*(37), 1-7, Article 37. <https://doi.org/10.1186/s13741-023-00328-w>

Alekaw, H., Derebe, D., Melese, W. M., & Yismaw, M. B. (2022). Antibiotic prescription pattern, appropriateness, and associated factors in patients admitted to pediatric wards of Tibebe Ghion Specialized Hospital, Bahir Dar, North West Ethiopia. *Infection and Drug Resistance*, *15*, 6659-6669. <https://doi.org/10.2147/IDR.S380897>

Amare, F., Gashaw, T., Sisay, M., Baye, Y., & Tesfa, T. (2021). The appropriateness of ceftriaxone utilization in government hospitals of Eastern Ethiopia: A retrospective evaluation of clinical practice. *SAGE Open Medicine*, *9*(2), 1-7. <https://doi.org/10.1177/20503121211051525>

Anteneh, D. A., Kifle, Z. D., Mersha, G. B., & Ayele, T. T. (2021). Appropriateness of antibiotics use and associated factors in hospitalized patients at University of Gondar Specialized Hospital, Amhara, Ethiopia: prospective follow-up study. *INQUIRY: The Journal of Health Care Organization, Provision, and Financing*, *58*(4), 1-9. <https://doi.org/10.1177/00469580211060744>

Asefa, L., Bayissa, G., & Abera, Z. (2016). Antibiotics use evaluation for pediatrics at nekemte referral hospital, East Wollega Zone, Oromia Region, West Ethiopia. *World Journal of Medical Sciences*, *13*(1), 17-26. <https://www.researchgate.net/publication/303410951>

Ayele, A. A., Gebresillassie, B. M., Erku, D. A., Gebreyohannes, E. A., Demssie, D. G., Mersha, A. G., & Tegegn, H. G. (2018). Prospective evaluation of Ceftriaxone use in medical and emergency wards of Gondar university referral hospital, Ethiopia. *Pharmacology Research and Perspectives*, *6*(1), 1-7. <https://doi.org/10.1002/prp2.383>

Ayinalem, G., Gelaw, B., Belay, A., & Linjesa, J. (2013). Drug use evaluation of ceftriaxone in medical ward of Dessie Referral Hospital, North East Ethiopia. *International Journal of Basic & Clinical Pharmacology*, *2*(6), 711-717. <https://doi.org/10.5455/2319-2003.ijbcp20131208>

Bantie, L. (2014). Drug use evaluation (DUE) of Ceftriaxone injection in the in-patient wards of Felege Hiwot Referral Hospital (FHRH), Bahir Dar, North Ethiopia. *International Journal of Pharma Sciences*, *4*(4), 671-676. <https://www.researchgate.net/publication/264536264>

Belayneh, Y. M., Amberbir, G., & Agalu, A. (2018). A prospective observational study of drug therapy problems in medical ward of a referral hospital in northeast Ethiopia. *BMC health services research*, *18(1)*, 1-7. <https://doi.org/10.1186/s12913-018-3612-x>

Birarra, M. K., Mekonnen, G. B., Gelayee, D. A., Assimamaw, N. T., & Kifle, Z. D. (2022). Drug dose adjustment in patients with renal impairment attending a specialized referral hospital, Northwest Ethiopia. *Metabolism Open*, *16*(9), 1-6. <https://doi.org/10.1016/j.metop.2022.100211>

Debela, G. A., Tesfaye, B. T., & Yizengaw, M. A. (2022). Risk Factors for Inappropriate Antimicrobial Therapy Among Patients with Hospital-Acquired Infection at Jimma Medical Center: A Prospective Observational Study. *Infection and Drug Resistance*, *15*, 837-850. <https://doi.org/10.2147/IDR.S349358>

Fekadu, G., Sado, E., Chennupati, S. V., & Bayisa, G. (2017). Evaluation of antibiotics use and prescribing error among hospitalized pediatric patients in Nekemte Referral Hospital, Ethiopia. *Journal of Applicable Chemistry*, *6*(4), 1-14. <https://www.researchgate.net/publication/318884247>

Firomsa, B., Ginenus, F., Kumera, B., Dinka, D., & Jiregna, S. (2021). Drug-related problems among patients with infectious disease admitted to medical wards of Wollega University Referral Hospital: Prospective observational study. *SAGE Open Medicine*, *9*(12), 1-8. <https://doi.org/10.1177/2050312121989625>

Firomsa, B., Kumera, B., & Dinka, D. (2019). Retrospective drug use evaluation of antibiotics in pediatric ward of Shambu general hospital, Oromia Region, West Ethiopia. *International Journal Of Modern Pharmaceutical Research*, *3*(2), 31-41. <https://www.researchgate.net/publication/332298982>

Firomsa, B., Tesfaye, T., Efrem, N., & Ginenus, F. (2021). Magnitude and determinants of drug-related problems among patients admitted to medical wards of southwestern Ethiopian hospitals: A multicenter prospective observational study. *PLoS One*, *16*(3), 1-12. <https://doi.org/10.1371/journal.pone.0248575>

Garedow, A. W., & Tesfaye, G. T. (2022). Evaluation of Antibiotics Use and its Predictors at Pediatrics Ward of Jimma Medical Center: Hospital Based Prospective Cross-sectional Study. *Infection and Drug Resistance*, *15*, 5365-5375. <https://doi.org/10.2147/IDR.S381999>

Gashaw, M., Berhane, M., Bekele, S., Melaku, T., Lemmi, G., Chelkeba, L., Wakjira, T., Tesfaw, G., Mekonnen, Z., Kroidl, A., Wieser, A., Froeschl, G., Seeholzer, T., Ali, S., & Gudina, E. K. (2025). Antibiotic Use Patterns at Jimma Medical Center in Southwest Ethiopia: A Call for Local Antibiogram-Guided Prescription. *Journal of Clinical Medicine*, *14*(7), 1-15. <https://doi.org/10.3390/jcm14072413>

Gebremichael, T. G., Gebreyesus, H. H., & Gebremariam, A. (2019). Assessment of the appropriateness of ceftazidime use in a tertiary teaching hospital, Northern Ethiopia. *Drug, Healthcare and Patient Safety*, *11*, 115-123. <https://doi.org/10.2147/DHPS.S226505>

Gebretekle., G. B., Mariam., D. H., Taye., W. A., Fentie., A. M., AmogneDegu., W., Alemayehu., T., Beyene., T., Libman., M., Fenta., T. G., Yansouni., C. P., & Semret., M. (2020). Half of Prescribed Antibiotics Are Not Needed: A Pharmacist-Led Antimicrobial Stewardship Intervention and Clinical Outcomes in a Referral Hospital in Ethiopia. *Front Public Health*, *8*, 1-11. <https://doi.org/10.3389/fpubh.2020.00109>

Geresu, G. D., Yadesa, T. M., & Deresa, B. (2018). Drug Use Evaluation of Ceftriaxone in Medical Ward of Mizan Aman General Hospital, Bench Maji Zone, South Western Ethiopia. *Journal of Bioanalysis & Biomedicine*, *10*(6), 123-131. <https://doi.org/10.4172/1948-593x.1000221>

Gidey, K., Aregawi, S. G., Hailu, B. Y., Asgedom, S. W., & Niriayo, Y. L. (2024). Antimicrobial Use-Related Problems Among Hospitalized Pediatric Patients: A Prospective Observational Study. *Infection and Drug Resistance*, *17*, 119-130. <https://doi.org/10.2147/IDR.S433677>

Gidey, K., Gebre-Samuel, N., Molla, F., Abrha, S., Wondimu, A., Assen, A., Melkam, W., & Pattnaik, G. (2015). Drug dose adjustment practices in patients with renal impairment at ayder referral hospital, Mekelle, northern Ethiopia. *International Journal of Pharmaceutical Sciences Review and Research*, *30*(2), 153-157. <https://globalresearchonline.net/journalcontents/v30-2/27.pdf>

Gube, A., Gonfa, R., & Tadesse, T. (2017). Evaluation of antibiotic use in medical Ward of Fitche District hospital, north Showa zone, Oromia region, Ethiopia. *Advances in Pharmacoepidemiology & Drug Safety*, *6*(3), 1-4. <https://doi.org/10.4172/2167-1052.1000217>

Habteweld, H. A., Yimam, M., Tsige, A. W., Wondmkun, Y. T., Endalifer, B. L., & Ayenew, K. D. (2023). Surgical site infection and antimicrobial prophylaxis prescribing profile, and its determinants among hospitalized patients in Northeast Ethiopia: a hospital based cross-sectional study. *Scientific Reports*, *13*(1), 1-9. <https://doi.org/10.1038/s41598-023-41834-7>

Jambo, A., Edessa, D., Adem, F., & Gashaw, T. (2023). Appropriateness of antimicrobial selection for treatment of pneumonia in selected public hospitals of Eastern Ethiopia: A cross-sectional study. *SAGE Open Medicine*, *11*(2), 1-11. <https://doi.org/10.1177/20503121231163792>

Jifar, W. W., Adugna, D., Gadisa, B., Debele, G. R., & Admasu, T. T. (2022). Retrospective Drug use Evaluation of Ceftriaxone In Resource Limited Setting In Case Of Bedele General Hospital, Ethiopia. *Research square* 1-15. <https://doi.org/10.21203/rs.3.rs-2315263/v1>

Kebede, H. K., Tefera, K., Azeb, W., Yemsrach, W., & Ayda, R. (2018). Assessment of ceftriaxone use in Eastern Ethiopian Referral Hospital: A retrospective study. *Journal of Infectious Diseases and Medical Microbiology*, *2*(3), 26-29. <http://www.alliedacademies.org/journal-infectious-diseases-medical-microbiology/>

Kefale, B., Tegegne, G. T., Degu, A., Molla, M., & Kefale, Y. (2020). Surgical site infections and prophylaxis antibiotic use in the surgical ward of public hospital in Western Ethiopia: a hospital-based retrospective cross-sectional study. *Infection and Drug Resistance*, *15*(13), 3627-3635. <https://doi.org/10.2147/IDR.S281097>

Ketema, D., Mekonnen, A., & Demissie, D. (2019). Antibiotics Prescription Practice for Inpatients Clients at St. Paul’s Hospital Mellinium Medical College in Addis Ababa, Ethiopia: Cross Sectional Study. *J Pharma Care Health Sys*, *6*(204), 2376-0419.1000204.

Mama, M., Mamo, A., Usman, H., Hussen, B., Hussen, A., & Morka, G. (2020). Inappropriate antibiotic use among inpatients attending Madda Walabu University Goba Referral Hospital, Southeast Ethiopia: implication for future use. *Infection and Drug Resistance*, *12*(13), 1403-1409. <https://doi.org/10.2147/IDR.S251151>

Mehari, K. (2017). *Evaluation of Ceftriaxone Utilization and Prescriber’s Opinion at Armed Forces Referral and Teaching Hospital, Addis Ababa, Ethiopia* Addis Ababa University].

Mengesha, Y., & Mohammed, S. (2025). Antibiotic Prescription Trends Among Pediatric Patients in Dessie Comprehensive Specialized Hospital, Ethiopia. *Sage Open Pediatrics*, *12*(1), 1-7. <https://doi.org/10.1177/30502225251315062>

Moges, G., Belete, L., Mengesha, Y., & Ahmed, S. (2020). Evaluation of surgical antimicrobial prophylaxis and incidence of surgical site infection at borumeda hospital, northeast ethiopia: Retrospective cross-sectional study. *Drug, Healthcare and Patient Safety*, *12*, 257-268. <https://doi.org/10.2147/DHPS.S280442>

Mohamoud, S. A., Yesuf, T. A., & Sisay, E. A. (2016). Utilization assessment of surgical antibiotic prophylaxis at Ayder Referral Hospital, Northern Ethiopia. *Journal of Applied Pharmacy*, *8*(02), 1-5. <https://doi.org/10.4172/1920-4159.1000220>

Muhammed, O. S., & Nasir, B. B. (2020). Drug use evaluation of ceftriaxone in Ras-Desta Memorial General Hospital, Ethiopia. *Drug, Healthcare and Patient Safety*, *12*, 161-168. <https://doi.org/10.2147/DHPS.S260364>

Mulat, A. (2023). *Magnitude of Appropriate Use of Surgical Antibiotic Prophylaxis and Associated Factors Among Surgical Operated Patients in Tibebe Ghion Specialized Hospital, Bahirdar, Northwest Ethiopia* Bahirdar University ].

Niriayo, Y. L., Ayalneh, M., Demoz, G. T., Tesfay, N., & Gidey, K. (2023). Antimicrobial use related problems and determinants in surgical ward of Ethiopian tertiary hospital. *PLoS One*, *18*(12), 1-14. <https://doi.org/10.1371/journal.pone.0296284>

Sewagegn, N., Ayichew, G. M., Miskir, A., Degarege, A., Mohammed, O., & Andualem, T. (2017). Evaluation of Ceftriaxone Use for Hospitalized Patients in Ethiopia: The Case of a Referral Hospital. *Annals of Case Reports*, *4*(4), 1-5. <https://doi.org/10.29011/2574-7754/100045>

Shegute, T., Hiruy, M., Hadush, H., & Gebremeskel, L. (2023). Ceftriaxone Use Evaluation in Western Zone Tigray Hospitals, Ethiopia: A Retrospective Cross-Sectional Study. *BioMed Research International*, *13*(11), 1-8. <https://doi.org/10.1155/2023/7688896>

Shimels, T., Bilal, A. I., & Mulugeta, A. (2015). Evaluation of Ceftriaxone utilization in internal medicine wards of general hospitals in Addis Ababa, Ethiopia: a comparative retrospective study. *Journal of Pharmaceutical Policy and Practice*, *8*, 1-8. <https://doi.org/10.1186/s40545-015-0047-1>

Shimels, T., & Fenta, T. G. (2016). Assessment of ceftriaxone utilization in different wards of the Federal Police Referral Hospital in Ethiopia: a retrospective study. *Ethiopian Pharmaceutical Journal*, *31*(2), 141-150. <https://doi.org/10.4314/epj.v31i2.6>

Sileshi, A., Tenna, A., Feyissa, M., & Shibeshi, W. (2016). Evaluation of ceftriaxone utilization in medical and emergency wards of Tikur Anbessa specialized hospital: A prospective cross-sectional study. *BMC Pharmacology and Toxicology*, *17*(7), 1-10. <https://doi.org/10.1186/s40360-016-0057-x>

Taressa, D., Teshome Sosengo, Jambo, A., Mathewos, E., Abdella, J., & Amare, F. (2021). Appropriateness of ceftriaxone prescription: A case of Haramaya hospital Eastern Ethiopia. *Journal of Basic and Clinical Pharmacy*, *12*(3), 1-10. <https://www.researchgate.net/publication/360498640>

Tassew, S. G., Woldu, M. A., Degu, W. A., & Shibeshi, W. (2020). Management of hospital-acquired infections among patients hospitalized at Zewditu memorial hospital, Addis Ababa, Ethiopia: A prospective cross-sectional study. *PLoS One*, *15*(04). <https://doi.org/10.1371/journal.pone.0231949>

Tefera, G. M., Feyisa, B. B., & Kebede, T. M. (2019). Antimicrobial use–related problems and their costs in surgery ward of Jimma University Medical Center: Prospective observational study [Review]. *PLoS One*, *14*(5), Article e0216770. <https://doi.org/10.1371/journal.pone.0216770>

Tessema, Z., Teshale, C., & Hawaze, S. (2014). A retrospective review of antibiotic utilization in adult medical wards of a primary care hospital in Ethiopia. *International journal of Pharmacy*, *4*(1), 56-62. <https://www.researchgate.net/publication/269332931>

Werede, A., Endalew, D., Habte, E., Derso, G., Desalegn, H., Mamo, S., Asrat, S., Hunde, T., & Hailu, Y. (2023). Drug Use Evaluation Study of Ceftriaxone in Ras Desta Damtew Memorial Hospital 2022 GC. *Biomed Journal of Science and Technical Research*, *50*(1), 41221-41226. <https://doi.org/10.26717/BJSTR.2023.50.007889>

Wondm, S. A., Dagnew, E. M., Abegaz, S. T., Kiflu, M., & Kebede, B. (2022). Burden, risk factors, and management of neutropenic fever among solid cancer patients in Ethiopia. *SAGE Open Medicine*, *10*(12), 1-12. <https://doi.org/10.1177/20503121221098236>

Yadesa, T. M., Gudina, E. K., & Angamo, M. T. (2015). Antimicrobial Use-Related Problems and Predictors among Hospitalized Medical In-Patients in Southwest Ethiopia: Prospective Observational Study. *PLoS One*, *10*(12), 1-9. <https://doi.org/10.1371/journal.pone.0138385>

Yehualaw, A., Taferre, C., Bantie, A. T., & Demsie, D. G. (2021). Appropriateness and pattern of antibiotic prescription in pediatric patients at Adigart General Hospital, Tigray, Ethiopia. *BioMed Research International*, *10*(1), 1-7. <https://doi.org/10.1155/2021/6640892>
